# Dysregulation of multiple metabolic networks related to brain transmethylation and polyamine pathways in Alzheimer disease: A targeted metabolomic and transcriptomic study

**DOI:** 10.1371/journal.pmed.1003012

**Published:** 2020-01-24

**Authors:** Uma V. Mahajan, Vijay R. Varma, Michael E. Griswold, Chad T. Blackshear, Yang An, Anup M. Oommen, Sudhir Varma, Juan C. Troncoso, Olga Pletnikova, Richard O’Brien, Timothy J. Hohman, Cristina Legido-Quigley, Madhav Thambisetty

**Affiliations:** 1 Clinical and Translational Neuroscience Section, Laboratory of Behavioral Neuroscience, National Institute on Aging, National Institutes of Health, Baltimore, Maryland, United States of America; 2 University of Mississippi Medical Center, Jackson, Mississippi, United States of America; 3 Brain Aging and Behavior Section, Laboratory of Behavioral Neuroscience, National Institute on Aging, National Institutes of Health, Baltimore, Maryland, United States of America; 4 Glycoscience Group, NCBES National Centre for Biomedical Engineering Science, National University of Ireland Galway, Galway, Ireland; 5 HiThru Analytics, Princeton, New Jersey, United States of America; 6 Johns Hopkins School of Medicine, Baltimore, Maryland, United States of America; 7 Duke University School of Medicine, Durham, North Carolina, United States of America; 8 Vanderbilt Memory & Alzheimer’s Center, Vanderbilt Genetics Institute, Vanderbilt University Medical Center, Nashville, Tennessee, United States of America; 9 Institute of Pharmaceutical Science, Kings College London, London, United Kingdom; 10 Steno Diabetes Center Copenhagen, Gentofte, Denmark; University of Cambridge, UNITED KINGDOM

## Abstract

**Background:**

There is growing evidence that Alzheimer disease (AD) is a pervasive metabolic disorder with dysregulation in multiple biochemical pathways underlying its pathogenesis. Understanding how perturbations in metabolism are related to AD is critical to identifying novel targets for disease-modifying therapies. In this study, we test whether AD pathogenesis is associated with dysregulation in brain transmethylation and polyamine pathways.

**Methods and findings:**

We first performed targeted and quantitative metabolomics assays using capillary electrophoresis-mass spectrometry (CE-MS) on brain samples from three groups in the Baltimore Longitudinal Study of Aging (BLSA) (AD: *n* = 17; Asymptomatic AD [ASY]: n = 13; Control [CN]: *n* = 13) (overall 37.2% female; mean age at death 86.118 ± 9.842 years) in regions both vulnerable and resistant to AD pathology. Using linear mixed-effects models within two primary brain regions (inferior temporal gyrus [ITG] and middle frontal gyrus [MFG]), we tested associations between brain tissue concentrations of 26 metabolites and the following primary outcomes: group differences, Consortium to Establish a Registry for Alzheimer’s Disease (CERAD) (neuritic plaque burden), and Braak (neurofibrillary pathology) scores. We found significant alterations in concentrations of metabolites in AD relative to CN samples, as well as associations with severity of both CERAD and Braak, mainly in the ITG. These metabolites represented biochemical reactions in the (1) methionine cycle (choline: lower in AD, *p* = 0.003; S-adenosyl methionine: higher in AD, *p* = 0.005); (2) transsulfuration and glutathione synthesis (cysteine: higher in AD, *p* < 0.001; reduced glutathione [GSH]: higher in AD, *p* < 0.001); (3) polyamine synthesis/catabolism (spermidine: higher in AD, *p* = 0.004); (4) urea cycle (N-acetyl glutamate: lower in AD, *p* < 0.001); (5) glutamate-aspartate metabolism (N-acetyl aspartate: lower in AD, *p* = 0.002); and (6) neurotransmitter metabolism (gamma-amino-butyric acid: lower in AD, *p* < 0.001). Utilizing three Gene Expression Omnibus (GEO) datasets, we then examined mRNA expression levels of 71 genes encoding enzymes regulating key reactions within these pathways in the entorhinal cortex (ERC; AD: *n* = 25; CN: *n* = 52) and hippocampus (AD: *n* = 29; CN: *n* = 56). Complementing our metabolomics results, our transcriptomics analyses also revealed significant alterations in gene expression levels of key enzymatic regulators of biochemical reactions linked to transmethylation and polyamine metabolism. Our study has limitations: our metabolomics assays measured only a small proportion of all metabolites participating in the pathways we examined. Our study is also cross-sectional, limiting our ability to directly test how AD progression may impact changes in metabolite concentrations or differential-gene expression. Additionally, the relatively small number of brain tissue samples may have limited our power to detect alterations in all pathway-specific metabolites and their genetic regulators.

**Conclusions:**

In this study, we observed broad dysregulation of transmethylation and polyamine synthesis/catabolism, including abnormalities in neurotransmitter signaling, urea cycle, aspartate-glutamate metabolism, and glutathione synthesis. Our results implicate alterations in cellular methylation potential and increased flux in the transmethylation pathways, increased demand on antioxidant defense mechanisms, perturbations in intermediate metabolism in the urea cycle and aspartate-glutamate pathways disrupting mitochondrial bioenergetics, increased polyamine biosynthesis and breakdown, as well as abnormalities in neurotransmitter metabolism that are related to AD.

## Abstract 2

Uma Mahajan and coworkers study metabolic perturbations in specific brain regions in Alzheimer disease.

## Author summary

**Why was this study done?:**

- A growing body of evidence suggests that Alzheimer disease (AD) may be associated with dysregulation of multiple metabolic pathways, and identifying novel molecular targets underlying AD pathogenesis is essential for developing effective AD treatments.
- Past studies have shown that abnormalities in choline-related biochemical pathways may be associated with AD pathogenesis, specifically the transmethylation, polyamine synthesis/catabolism and related pathways.
- Our study tested the hypothesis that dysregulation of choline-related biochemical pathways in the brain is associated with AD pathogenesis; we examined metabolites within biochemical reactions linked to transmethylation and polyamine synthesis/catabolism.

**What did the researchers do and find?:**

- We performed quantitative and targeted metabolomics on brain tissue samples (AD: *n* = 17; Asymptomatic AD [ASY]: *n* = 13; Control [CN]: *n* = 13) and transcriptomics from Gene Expression Omnibus data (entorhinal cortex [AD: *n* = 25; CN: *n* = 52] and hippocampus [AD: *n* = 29; CN: *n* = 56]) to identify aberrations across 6 biochemical reactions linked to the transmethylation and polyamine pathways: methionine cycle, transsulfuration and glutathione synthesis, polyamine synthesis and catabolism, urea cycle, glutamate-aspartate metabolism, and neurotransmitter metabolism.
- We found significant metabolite alterations associated with AD mainly in the inferior temporal gyrus (ITG) across all pathways tested, as well as associations between metabolite concentrations and severity of AD pathology.
- Complementing our metabolomics results, our transcriptomics analyses also revealed significant alterations in gene expression of key enzymatic regulators of biochemical reactions linked to transmethylation and polyamine metabolism.

**What do these findings mean?:**

- Our results implicate alterations in cellular methylation potential and increased flux in the transmethylation pathways, increased demand on antioxidant defense mechanisms, perturbations in intermediate metabolism in the urea cycle and aspartate-glutamate pathways disrupting mitochondrial bioenergetics, increased polyamine biosynthesis and breakdown, as well as abnormalities in neurotransmitter metabolism that are related to severity of AD pathology and the expression of clinical symptoms.
- This study adds to a comprehensive understanding of the metabolic basis of AD pathogenesis and provides insights into novel targets for disease-modifying therapies.
- The cross-sectional nature of the study limits our ability to directly test how AD progression may impact changes in metabolite concentrations or differential-gene expression.

## Introduction

A growing body of evidence suggests that Alzheimer disease (AD) is a pervasive metabolic disorder with dysregulation of multiple biochemical pathways that may be associated with both severity of AD pathology in the brain and eventual expression of disease symptoms [1–4]. An enhanced understanding of abnormal metabolism in AD is likely to provide insights into novel targets for disease-modifying therapies. While the predominant approach towards AD treatments has targeted amyloid-beta (Aβ), the repeated failures of clinical trials of anti-amyloid therapies highlights the pressing need to identify novel molecular targets that may underlie AD pathogenesis [5–7].

In recent studies, we have used both targeted and untargeted metabolomics methods in both brain and blood to study the role of abnormal metabolism in AD pathogenesis [8,9]. These studies have revealed dysregulation in brain glycolysis as well as perturbations in metabolic pathways derived from acetyl–coenzyme A (CoA), produced by oxidation of the end product of glycolysis—pyruvate. These pathways include fatty acid and phospholipid metabolism [9,10]. Broadly, our prior results suggest that abnormalities in glycolysis in AD may impact the supply of cytosolic acetyl-CoA, which can lead to perturbations in other intermediate metabolic pathways. In addition to synthesis of fatty acids and their subsequent incorporation into phospholipids, acetyl-CoA combines with choline in cholinergic neurons to form the neurotransmitter acetylcholine. Acetylcholine plays a critical role in learning and memory, and abnormalities in cholinergic neurotransmission are thought to underlie cognitive impairment in AD [11]. Furthermore, acetylcholinesterase inhibitors constitute the major class of drugs that are currently approved symptomatic treatments for AD [12]. Choline is also a key participant in one-carbon metabolism (OCM) through its oxidation product betaine and constitutes the head group of phosphatidylcholines in cell membranes. Abnormalities in both OCM and phosphatidylcholine metabolism have previously been implicated in AD [13,14].

In this study, we therefore tested the hypothesis that dysregulation of choline-related biochemical pathways in the brain are associated with AD pathogenesis. We a priori selected the transmethylation pathway, the primary source of one-carbon units, and the closely related polyamine synthesis/catabolism pathway, both of which have been implicated in cell survival and signaling cascades relevant to neurodegeneration [15–19]. We examined metabolites within biochemical reactions linked to transmethylation and polyamine synthesis/catabolism within the following six categories: methionine cycle, transsulfuration and glutathione synthesis, polyamine synthesis/catabolism, urea cycle, glutamate-aspartate metabolism, and neurotransmitter metabolism. We analyzed available concentrations of metabolites within these categories derived from targeted metabolomics assays of brain tissue samples from the autopsy cohort of the Baltimore Longitudinal Study of Aging (BLSA). These assays were performed using capillary electrophoresis-mass spectrometry (CE-MS), an approach distinct from our prior metabolomics studies that examined abnormalities in brain glycolysis [8] and fatty acid [10] and glycerophospholipid [9] metabolism in AD using flow injection analysis-mass spectrometry (FIA-MS/MS) and liquid chromatography-tandem mass spectrometry (LC-MS/MS). We then analyzed publicly available transcriptomic datasets to test whether gene expression of genetic regulators of these pathways were altered in brain regions where accumulation of AD pathology is known to trigger the onset of cognitive impairment.

## Methods

This study is reported as per the STROBE guideline (S1 Table).

### Participants

#### Quantitative brain metabolomics

The National Institute on Aging’s (NIA’s) BLSA is one of the longest running scientific studies of human aging in the United States. [20]. This observational study began in 1958 and includes longitudinal clinical, radiological, and laboratory evaluations on community-dwelling volunteer participants. Participants are assessed every two years, and starting in 2003, participants older than 80 years are assessed annually.

Our sample consisted of a subset of participants from the autopsy program of the BLSA [21]. The autopsy subsample is not significantly different from the overall BLSA cohort in terms of rates of dementia and clinical stroke [22]. Postmortem brains were examined by an expert neuropathologist to assess AD pathology. As described previously [23], the Consortium to Establish a Registry for Alzheimer’s Disease (CERAD) and Braak criteria were used to assess severity of AD pathology based on neuritic plaques [24] and neurofibrillary tangles [25], respectively. Autopsy participants were classified within three groups based on the following criteria: AD participants (*n* = 17) had a clinical diagnosis of either AD or mild cognitive impairment (MCI) due to AD within 1 year of death and a CERAD pathology score of >1 (i.e., CERAD B or C); control (CN) participants (*n* = 13) had normal cognition within 1 year of death and a CERAD pathology score ≤1 (i.e., CERAD 0 or A); and asymptomatic AD (ASY) participants (*n* = 13) had normal cognition within 1 year of death and a CERAD pathology score >1 (i.e., CERAD B or C). The ASY group has been described in detail previously [26] and represents individuals who at autopsy had brain pathology characteristic of AD but did not exhibit clinical symptoms of AD (i.e., cognitive impairment) during life as assessed by longitudinal neuropsychological assessments.

Written informed consent was obtained at each visit for all BLSA participants. The BLSA study protocol has ongoing approval from the Institutional Review Board of the National Institute of Environmental Health Science, National Institutes of Health (“Early Markers of Alzheimer’s Disease [BLSA],” Institutional Review Board number 2009–074).

#### Brain gene expression

Our gene expression data were downloaded from three publicly available microarray gene expression datasets (Gene Expression Omnibus [GEO]: GSE48350, GSE5281, and GSE84422). The datasets contained gene expression data on AD and CN brain samples representing 23 distinct brain regions, acquired on the Affymetrix U133 Plus2 array platform. As prior studies have suggested that the entorhinal cortex (ERC) and the hippocampus are primary regions of early AD pathology accumulation [27] that trigger cognitive impairment [28,29], we performed a focused analysis of gene expression data from the ERC (AD: *n* = 25 and CN: *n* = 52) and the hippocampus (AD: *n* = 29 and CN: *n* = 56).

### Brain tissue

Tissue samples from participants in three groups (AD: *n* = 17; ASY: *n* = 13; CN: *n* = 13) were extracted from the cortical surface of autopsied brains using a sterile 4-mm-diameter tissue punch from three regions: inferior temporal gyrus (ITG), middle frontal gyrus (MFG), and cerebellum (CB). The ITG and the MFG represent our primary regions of interest and represent specific a priori hypotheses regarding the relationship between metabolite levels and distinct features of AD pathology. The ITG was chosen as a region of early tau accumulation and neurofibrillary pathology, while the MFG was chosen as a site of Aβ deposition and neuritic plaque pathology [30,31]. We hypothesized that both the ITG and MFG would be sites of metabolic pathway dysregulation associated with specific features of AD pathology. On the other hand, the CB was chosen primarily as a control, as the CB represents a brain region that is relatively unaffected by AD pathology [32]. In this ancillary analysis, we hypothesized that the metabolic pathways we assessed would not be altered and we would not observe significant differences in metabolite concentration across groups (i.e., AD, ASY, CN). Brain tissue samples from the ERC and hippocampus were not available for metabolomic profiling. All tissue samples were stored at −80 °C until metabolomic assays.

### Metabolomic profiling

#### Sample preparation

Quantitative and targeted metabolomics assays were performed on brain tissue samples by capillary electrophoresis time-of-flight mass spectrometry (CE-TOFMS) with the operator blinded to diagnoses. Metabolite measurements were carried out at Human Metabolome Technologies, Tsuruoka, Japan. Approximately 50 mg of frozen brain tissue was plunged into 1.5 mL of 50% acetonitrile/Milli-Q water at 0 °C containing 20 μM and 5 μM of internal standard for the cation (methionine sulfone) and anion (10-camphorsulfonic acid) modes, respectively, as described previously [33,34]. The tissue was homogenized thrice at 1,500 rpm for 120 seconds using a tissue homogenizer (Microsmash MS100R, Tomy Digital Biology, Tokyo, Japan). The homogenate was centrifuged at 2,300*g* and 4 °C for 5 minutes. Subsequently, 800 μL of upper aqueous layer was transferred to a 5-kDa-cutoff filter (Human Metabolome Technologies, Tsuruoka, Japan) to remove proteins. The filtrate was dried using a centrifugal concentrator (Labconco Corporation, Kansas City, MO) and reconstituted with 50 μL of Milli-Q water for CE-MS analysis.

#### CE-TOFMS

CE-TOFMS was carried out using an Agilent CE Capillary Electrophoresis System equipped with an Agilent 6210 Time of Flight mass spectrometer, Agilent 1100 isocratic HPLC pump, Agilent G1603A CE-MS adapter kit, and Agilent G1607A CE-ESI-MS sprayer kit (Agilent Technologies, Waldbronn, Germany). The systems were controlled by Agilent G2201AA ChemStation software version B.03.01 for CE (Agilent Technologies, Waldbronn, Germany). The sample was injected at a pressure of 50 mbar for 10 seconds (approximately 10 nL) in cation analysis and 25 seconds (approximately 25 nL) in anion analysis. The spectrometer was scanned from mass-to-charge ratio (*m/z*) 50 to 1,000. Other conditions were as described previously by Soga and colleagues [35–37].

Peaks were extracted using automatic integration software ver. 2.13.0.8 (MasterHands, Keio University, Tsuruoka, Japan) [34] and were aligned according to *m/z* and migration time (MT). The relative area value was calculated by dividing the acquired peak area value by that of the internal standard and the sample volume [34, 38]. After peak picking, the MT drifts of all electropherograms in each sample were corrected based on those of the internal standards containing methionine sulfone and 10-camphorsulfonic acid by fitting quadratic functions. The peak matrix was matched against the annotation table of the in-house metabolomic library (Human Metabolome Technologies) described previously on the basis of their *m/z* and MTs [38]. The tolerance range for the peak annotation was configured at ±0.2 minutes for MT and ±10 ppm for *m/z*.

HMT’s in-house metabolite library contains 1,363 compounds, as described recently by Sasaki and colleagues [39]. A full listing of available compounds in the library is accessible through their recent publication at https://pubs.acs.org/doi/abs/10.1021/acs.analchem.8b02994.

CE-TOFMS analysis using the current system enables measurement of the concentrations of 130 major metabolites in each sample on the basis of their peak areas. Six-point calibration curves were previously derived using standard compounds for each metabolite reported herein to evaluate the linearity and detection limits for each analyte. In assays performed in the current study, we calculate the concentration of metabolites in singlicate using 1-point calibration of each standard compound in the linear range at 100 μM.

These metabolites represent glycolysis, pentose phosphate pathway, TCA cycle, neuro-signaling, and the urea cycle, as well as polyamine, creatine, purine, glutathione, nicotinamide, choline, and amino acid metabolism. Quantification is performed using the (M+H)+ or (M-H)− parent ion peak area for each metabolite compared to the same parent peak in the standard solution. Concentration is only reported if the measured area is above a signal-to-noise ratio (S/N) of 5 and the peak area is within the linear range of the standard curve [39]. Coefficients of variation (CVs) for repeat technical samples using this methodology were less than 10%, as reported previously by Soga and colleagues [40]. S2 Table includes a list of all 27 metabolites in the transmethylation and polyamine pathways that were studied in this report, including their class, ion type, *m/z*, MT, S/N, and limit of detection data.

Of the 130 metabolites that could be quantified in brain tissue samples, we a priori identified 27 metabolites that represented reactions within the transmethylation and polyamine pathways, which were the main focus of our study. In order to enhance ease of interpretation of our results, we grouped these metabolites into six categories based on the primary biochemical reactions in which they participate. These categories and their constituent metabolites (26 metabolites after exclusion of spermine, based on criteria described in Statistical Analyses) include the following:

1. Methionine cycle: betaine, choline, creatine, methionine (Met), methionine sulfoxide, S-adenosylmethionine (SAM), S-adenosylhomocysteine (SAH), and symmetric dimethylarginine (SDMA).
2. Transsulfuration and glutathione synthesis: reduced glutathione (GSH), oxidized glutathione disulfide (GSSG), cysteine (Cys), and cystathionine.
3. Polyamine synthesis and catabolism: putrescine and spermidine.
4. Urea cycle: arginine, argininosuccinic acid, citrulline, N-acetylglutamate (NAG), ornithine, and urea.
5. Glutamate-aspartate metabolism: alanine, aspartate, glutamine (Gln), and glutamate (Glu).
6. Neurotransmitter metabolism: Gamma-amino-butyric acid (GABA) and N-acetylaspartate (NAA).

### Gene expression

Because accumulation of pathology in the ERC and hippocampus is believed to trigger the onset of AD symptoms [27–29], we were also interested in examining whether differential expression of genes regulating reactions within the six biochemical categories described above could be detected in these regions.

We generated an a priori list of genes known to encode enzymes regulating individual reactions within the same six biochemical categories described above for the classification of assayed metabolites.

In order to confirm that regional gene expression in the ERC and hippocampus represented measures relevant to those in the ITG and MFG where our metabolomics assays were performed, we also examined correlations between gene expression in the ERC and hippocampus with gene expression in the ITG and MFG.

### Statistical analyses

The overall study design was developed conceptually prior to the start of data analyses in July 2018 and is similar to the design implemented in our prior papers [9,10]. The study did not have a prespecified analysis plan. Based on reviewer recommendations, we updated our analytic approach from independent Kruskal-Wallis tests to cluster-based generalized linear mixed models to more appropriately account for correlations between metabolites within our a priori–defined metabolite categories, as well as to better adjust for multiple comparisons.

As in our previous metabolomics analyses [9], metabolites with greater than 30% of values missing were dropped from all analyses. From the a priori–defined 27 principal metabolites in the transmethylation and polyamine pathways, 1 metabolite (spermine) was dropped due to missingness >30%. For the remaining metabolites (i.e., 26 in total), values indicated as less than the limit of detection were imputed as the lowest detectable value divided by 2 (1.15% of metabolites imputed on average).

R-Studio 1.1.453 and Stata 16.0 were used for analyses. Statistical significance was determined using an alpha of 0.05. While we present our findings as exploratory, multiple comparisons controlling for Benjamini-Hochberg false discovery rate (FDR = 0.05) [41] and Bonferroni family-wise error rate (FWER = 0.05) were conducted in response to reviewers suggestions; Bonferroni techniques are known to be markedly conservative and can be inappropriate when control regions are included [42]. Both multiple comparison methods corrected for 52 comparisons (26 metabolites × 2 primary brain regions of interest [ITG and MFG]) that were a priori specified based on their vulnerability to distinct AD pathologies. Differences across the three groups (AD, ASY, and CN) in sex, APOE ε4 carrier status (Fisher-exact test), age, and postmortem interval (PMI) (Kruskal-Wallis test) were examined.

The main analysis of interest was to assess differences in metabolite levels across groups. We used linear mixed-effects models on each of the a priori–defined metabolite categories (i.e., clusters) within each of the two primary brain regions (ITG and MFG) as well as the secondary control region (CB), with log2-transformed concentrations as the dependent variable, group as the fixed effect, unstructured within-subject residual correlations, and Huber-White robust variance estimates. Using the same analytic approach, we also examined brain tissue metabolite associations with Braak and CERAD pathology scores for the primary brain regions only (i.e., ITG and MFG) based on reviewer recommendations to look at associations with pathology only for brain regions with significant group differences. Sensitivity analyses using generalized estimating equation (GEE) clustered analyses and nonparametric Spearman correlations with independent Kruskal-Wallis tests gave similar results.

For gene expression data, we first normalized the samples using Robust Multi-array Average (RMA) [43] with the Brainarray ENTREZG (version 22) custom CDF [44]. We used the GEO datasets GSE48350, GSE5281, and GSE8442, and we used the GEO dataset name as the batch covariate for all further statistical analyses. We then tested for differences in sex, age, and batch between AD and CN samples using the one-way ANOVA test. The R package *limma* [45] was used to test each gene univariately for differences between AD and CN samples, controlling for the effects of sex, age, and batch and adjusting for multiple comparisons using the FDR (set at *p* = 0.05) [41] accounting for all 20,414 genes on the Affymetrix U133 Plus 2.0 array used in the GEO datasets. We summarized results of sex, age, and batch-corrected fold changes indicating whether genes were differentially expressed in AD versus CN samples and visualized significant (either in the hippocampus and/or ERC) results using a heatmap. Red and green represent increased and reduced expression, respectively, of the gene in AD versus CN samples (i.e., red indicates that the gene had increased expression in AD samples compared to CN samples, and green indicates that the gene has reduced expression in AD samples compared to CN samples).

Analytic code used in statistical analyses are available upon request to the corresponding author.

## Results

### Participants

The demographic characteristics of BLSA participants in the autopsy cohort sample are summarized in Table 1. As reported previously [9], the three groups did not differ significantly in age at death, sex, apolipoprotein *E* (*APOE* ε4) carrier status, or PMI. Demographic characteristics of participants whose gene expression data were available in the three GEO datasets are included in Table 2. In the GEO data, AD participants were significantly older than CN participants (*p* < 0.05). BLSA participants and GEO participants did not differ by sex; BLSA participants were significantly older than GEO participants (*p* < 0.05).

**Table 1:** Demographic characteristics of the BLSA autopsy sample.

Characteristics	Total	CN	ASY	AD	*p*-value
***n***	43	13	13	17	-
**Female (%)**	37.2	23.1	38.5	47.1	0.401
**Age (mean, SD)**	86.118 (9.842)	82.427 (11.510)	88.218 (8.041)	87.411 (9.468)	0.380
***APOE* ε4 allele (%)**	23.3	23.1	23.1	23.5	>0.999
**PMI (hours) (mean, SD)**	15.179 (6.760)	16.818 (6.0467)	15.042 (8.677)	14.156 (5.750)	0.715

Differences in sex and *APOE* ε4 allele were tested with the Fisher test, and differences in age and PMI were tested with the Kruskal-Wallis test.

Abbreviations: AD, Alzheimer disease; *APOE* ε4, apolipoprotein *E*; ASY, asymptomatic AD; BLSA, Baltimore Longitudinal Study of Aging; CN, Control; PMI, postmortem interval

**Table 2:** Demographics of individuals from GEO data.

Brain Region and Characteristics	AD	CN	Total	*p*-value
**ERC**	25	52	77	
Female (%)	56.0	40.4	45.5	0.228
Age (mean, SD)	86.12 (5.718)	62.288 (24.526)	70.026 (23.241)	<0.001*
**Hippocampus**	29	56	85	
Female (%)	48.3	41.1	43.5	0.645
Age (mean, SD)	81.241 (7.945)	66.464 (23.766)	71.506 (20.989)	0.010*
**Total**	54	108	162	

*Indicates *p*-value <0.05 for global group differences across demographic characteristics.

Differences in sex were tested with the Fisher test, and differences in age were tested with the Kruskal-Wallis test.

Abbreviations: AD, Alzheimer disease; CN, control; ERC, entorhinal cortex; GEO, Gene Expression Omnibus

### Brain tissue metabolite concentrations and their associations with AD

S3 Table indicates regional brain tissue concentrations (picomoles per milligram of brain tissue) of assayed metabolites by group.

**Fig 1:**
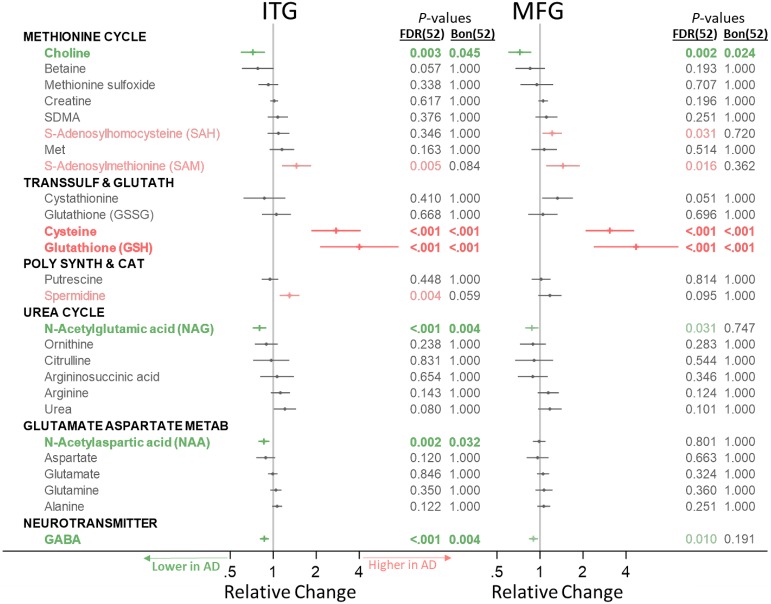
Group differences in category-specific metabolite concentrations. Metabolites in green or red (not bold) were significant after FDR correction for 52 comparisons; metabolites in green or red (bold) were also significant after Bonferroni correction for 52 comparisons. Differences in metabolite concentration across groups were tested using linear mixed-effects models for each metabolite category. AD, Alzheimer disease; Bon, Bonferroni; FDR, false discovery rate; Glutamate Aspartate Metab, glutamate-aspartate metabolism; ITG, inferior temporal gyrus; MFG, medial temporal gyrus; Poly Synth & Cat, polyamine synthesis and catabolism; Transsulf and Glutath, transsulfuration and glutathione synthesis.

**Fig 2:**
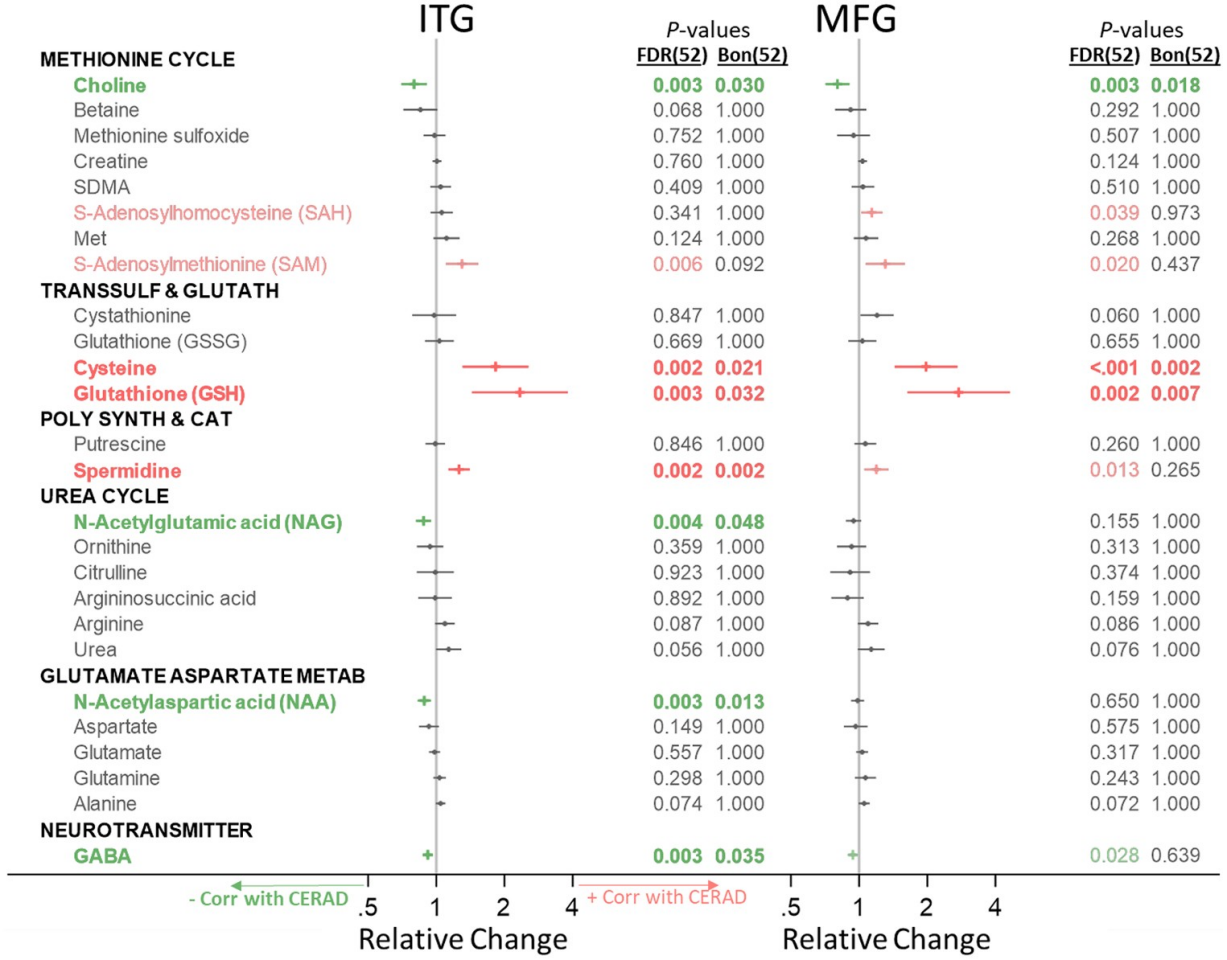
Associations between category-specific metabolites and neuritic plaque burden (CERAD). Metabolites in green or red (not bold) were significant after FDR correction for 52 comparisons; metabolites in green or red (bold) were also significant after Bonferroni correction for 52 comparisons. Associations with pathology were tested using linear mixed-effects models for each metabolite category. Bon, Bonferroni; CERAD, Consortium to Establish a Registry for Alzheimer’s Disease; FDR, false discovery rate; Glutamate Aspartate Metab, glutamate-aspartate metabolism; ITG, inferior temporal gyrus; MFG, medial temporal gyrus; Poly Synth & Cat, polyamine synthesis and catabolism; Transsulf and Glutath, transsulfuration and glutathione synthesis.

**Fig 3:**
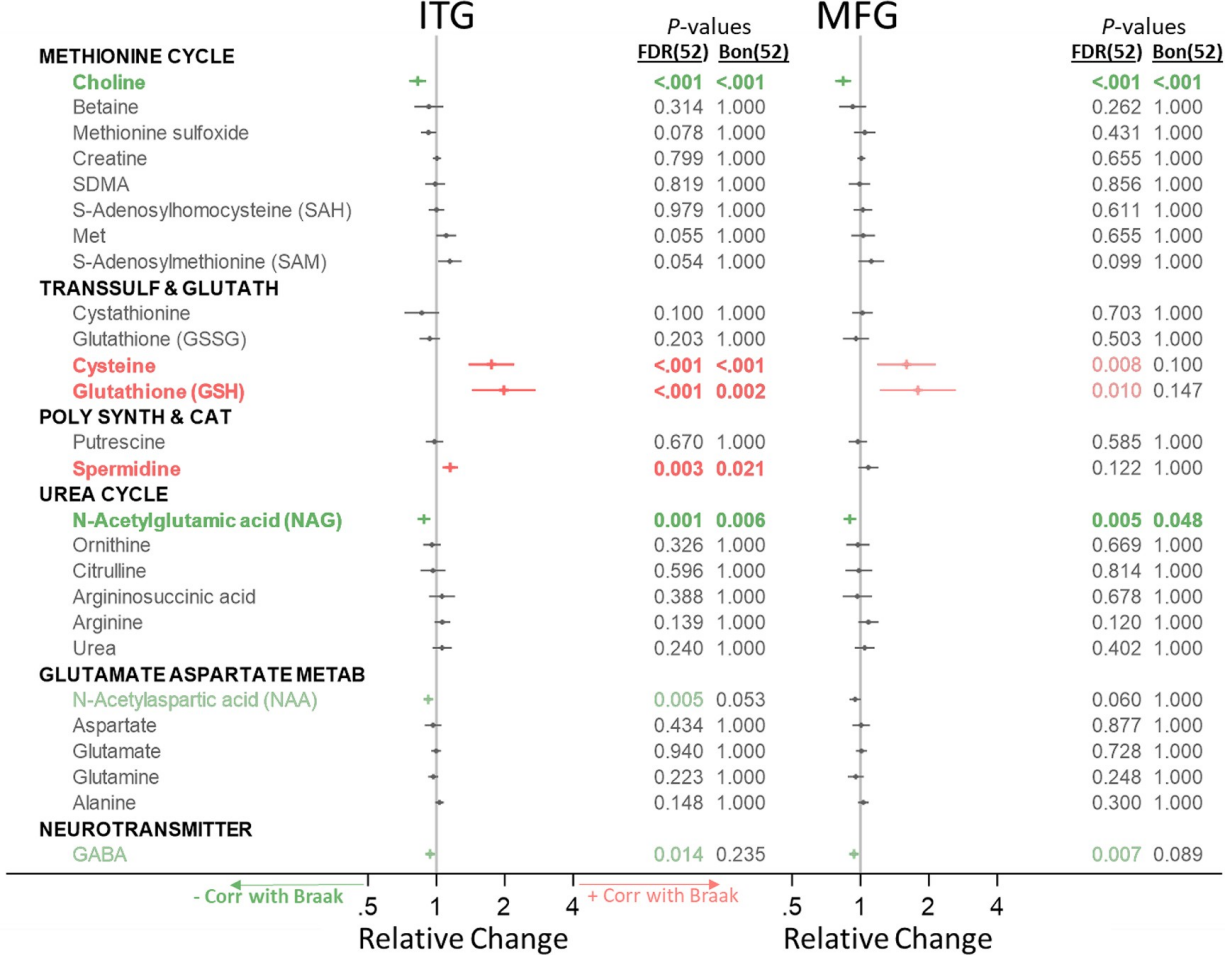
Associations between category-specific metabolites and neurofibrillary pathology (Braak). Metabolites in green or red (not bold) were significant after FDR correction for 52 comparisons; metabolites in green or red (bold) were also significant after Bonferroni correction for 52 comparisons. Associations with pathology were tested using linear mixed-effects models for each metabolite category. Bon, Bonferroni; CERAD, Consortium to Establish a Registry for Alzheimer’s Disease; FDR, false discovery rate; Glutamate Aspartate Metab, glutamate-aspartate metabolism; ITG, inferior temporal gyrus; MFG, medial temporal gyrus; Poly Synth & Cat, polyamine synthesis and catabolism; Transsulf and Glutath, transsulfuration and glutathione synthesis.

Below, for our main analysis, we indicate significant metabolite concentration differences across groups by category (Fig 1) and include forest plots visualizing the direction of change and effect size. We additionally indicate associations between metabolite concentration and neuritic plaque burden (i.e., CERAD scores) (Fig 2) and neurofibrillary pathology (i.e., Braak scores) (Fig 3). Metabolites that are significantly altered after FDR correction are colored in green (indicating a lower metabolite concentration in AD or negative association with AD pathology) or red (indicating a higher metabolite concentration in AD or positive association with AD pathology) in Figs 1–3, and metabolites that remain statistically significant after Bonferroni correction are additionally indicated in bold. We visualize results specifically for the main results—ITG group differences—in pathway-specific figures. As suggested by the reviewers, we include both FDR- and Bonferroni-corrected *p*-values in all tables. In results described below in the text as well as pathway-specific figures, we report FDR-corrected *p*-values and indicate whether metabolites were additionally also significant after Bonferroni correction with an asterisk. We report ancillary analyses within the CB in S4 Table.

As reported previously, the three groups (AD, ASY, and CN) differed significantly in the severity of neuritic plaque and neurofibrillary pathology, as measured by CERAD and Braak scores, respectively (*p* < 0.0001), with the AD group showing the highest, ASY group showing intermediate, and CN group showing the lowest levels of pathology [8].

#### Methionine cycle (Fig 4)

**Fig 4:**
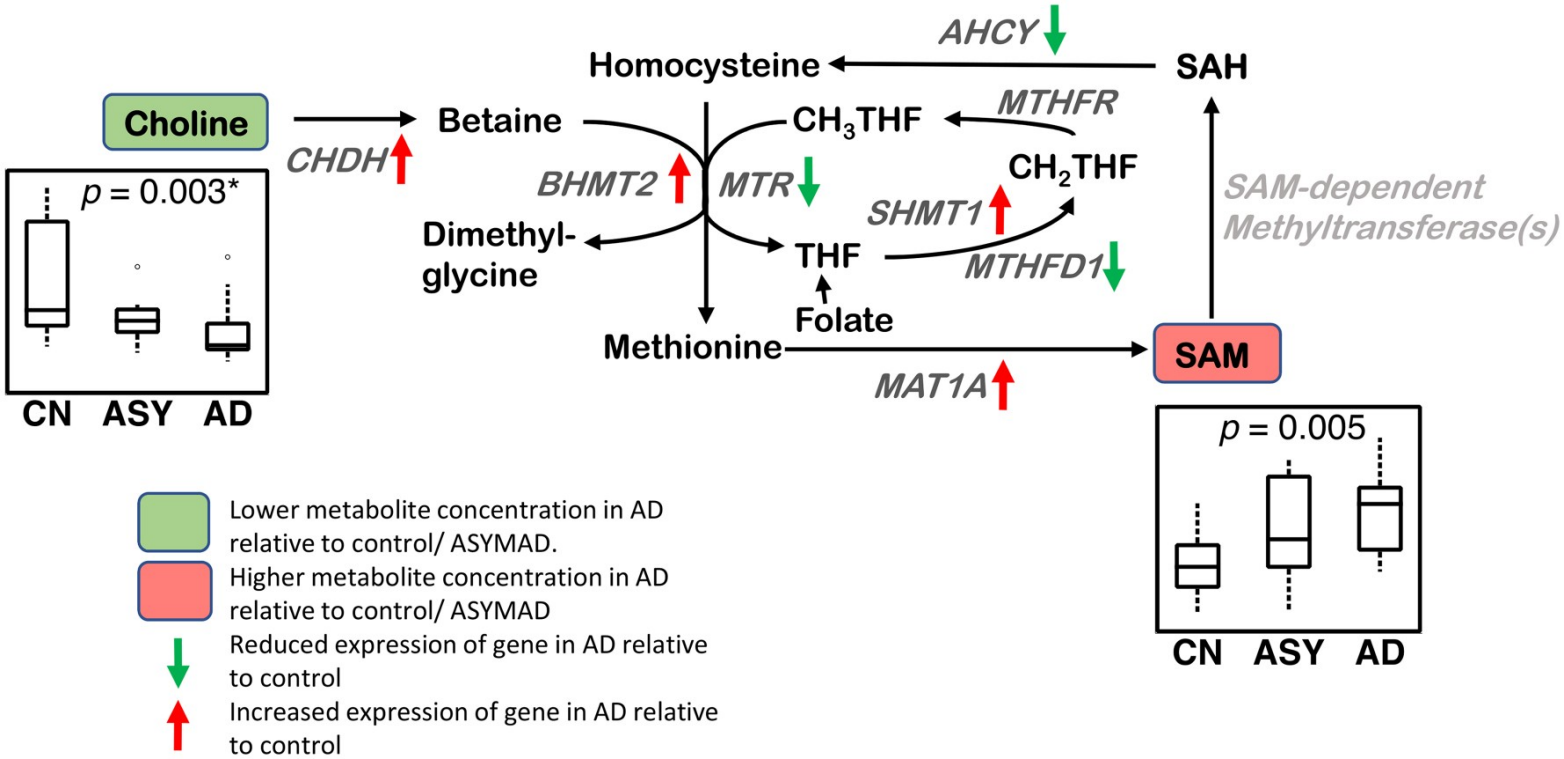
Methionine cycle. Differential brain concentrations of metabolites and differential gene expression of enzymatic regulators are observed in the methionine cycle in AD. Lower concentration of choline and higher concentration of SAM are observed in AD in the ITG. CHDH, BHMT2, SHMT1, and MAT1A genes have increased expression and MTR, AHCY, and MTHFD1 genes have reduced expression in the hippocampus/ERC in AD compared to CN. Sample size: AD = 17, ASY = 13, CN = 13; *p*-values indicate significance after FDR correction for multiple comparisons. Genes in light gray indicate expression data were not available. An asterisk (*) indicates metabolite was also significant after Bonferroni correction. AD, Alzheimer disease; AHCY, adenosylhomocysteinase; ASY, asymptomatic AD; BHMT2, betaine-homocysteine s-methyltransferase 2; CHDH, choline dehydrogenase; CN, Control; ERC, entorhinal cortex; FDR, false discovery rate; ITG, inferior temporal gyrus; MAT1A, methionine adenosyltransferase 1A; MTHFD1, methylenetetrahydrofolate dehydrogenase, cyclohydrolase, and formyltetrahydrofolate synthetase 1; MTHFR, methylenetetrahydrofolate reductase; MTR, 5-methyltetrahydrofolate-homocysteine methyltransferase; SAH, S-adenosyl homocysteine; SAM, S-adenosyl methionine; SHMT1, serine hydroxymethyltransferase 1.

We observed a lower concentration of choline (ITG: *p* = 0.003*; MFG: *p* = 0.002*) in AD, as well as associations between a lower concentration of choline and greater neuritic plaque burden (ITG: *p* = 0.003*; MFG: *p* = 0.003*) and greater neurofibrillary pathology (ITG: p < 0.001*; MFG: p < 0.001*). We observed a higher concentration of SAM (ITG: *p* = 0.005; MFG: *p* = 0.016) in AD as well as associations between higher concentrations of SAM and greater neuritic plaque burden (ITG: *p* = 0.006; MFG: *p* = 0.020). We also observed a higher concentration of SAH (MFG: *p* = 0.031) in AD.

#### Transsulfuration and glutathione synthesis (Fig 5)

**Fig 5:**
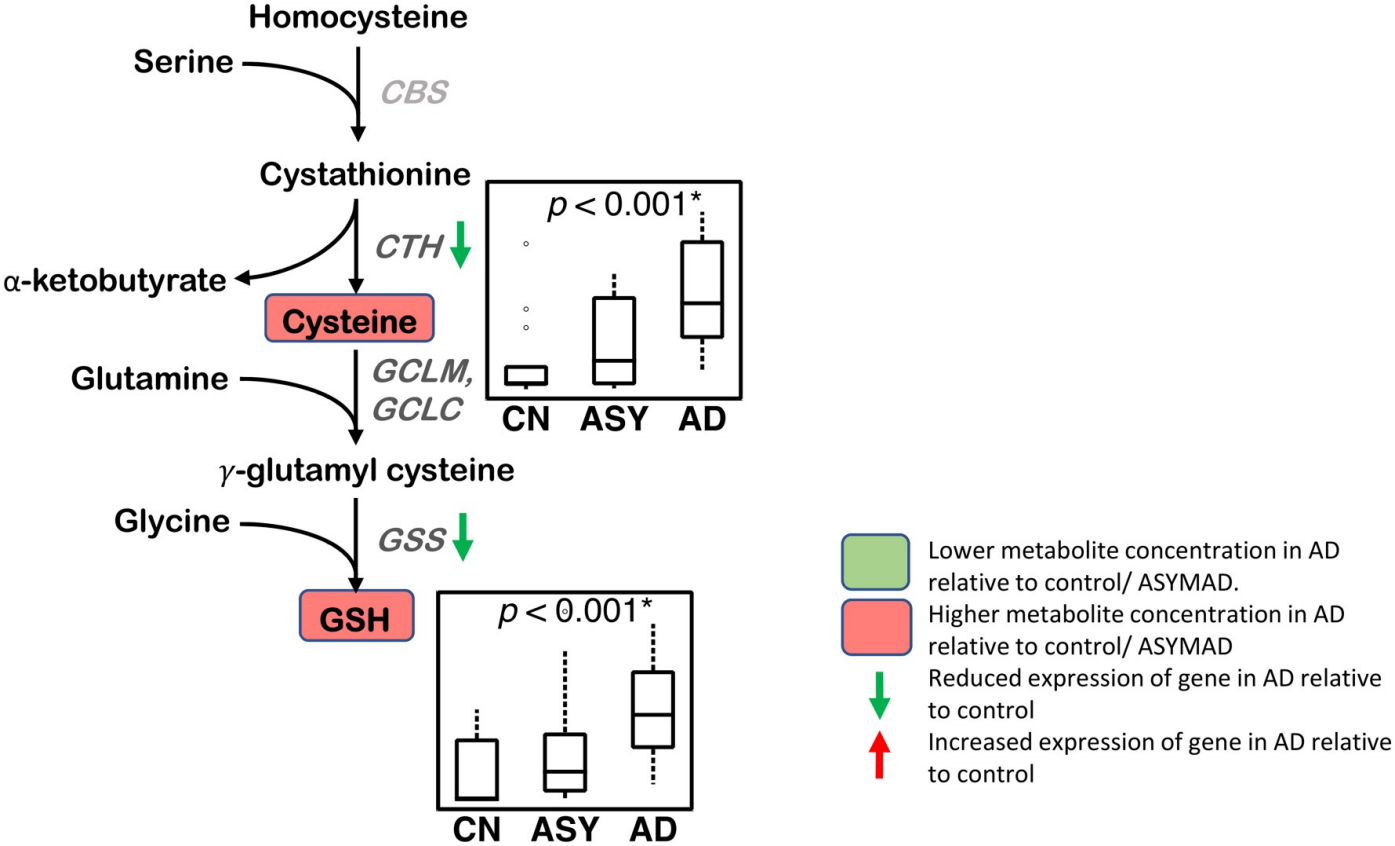
Transsulfuration and glutathione synthesis. Higher concentrations of cysteine and GSH are observed in AD in the ITG. Differential gene expression of enzymatic regulators of transsulfuration and glutathione synthesis are observed in the hippocampus/ERC in AD relative to controls. CTH and GSS genes have reduced expression in the hippocampus/ERC in AD compared to CN. Sample size: AD = 17, ASY = 13, CN = 13; *p*-values indicate significance after FDR correction for multiple comparisons. An asterisk (*) indicates metabolite was also significant after Bonferroni correction. AD, Alzheimer disease; ASY, asymptomatic AD; CBS, cystathione β-synthase; CN, Control; CTH, cystathionase; ERC, entorhinal cortex; FDR, false discovery rate; GCLC, *γ*-glutamyl cysteine ligase; GCLM, glutamate-cysteine ligase modifier subunit; GSH, reduced glutathione; GSS, GSH synthetase; ITG, inferior temporal gyrus.

We observed a higher concentration of cysteine (ITG: *p* < 0.001*; MFG: *p* < 0.001*) in AD, as well as associations between higher concentrations of cysteine and greater neuritic plaque burden (ITG: *p* = 0.002*; MFG: *p* < 0.001*) and greater neurofibrillary pathology (ITG: *p* < 0.001*; MFG: *p* = 0.008). We observed a higher concentration of GSH (ITG: *p* < 0.001*; MFG: *p* < 0.001*) in AD, as well as associations between higher concentrations of GSH and greater neuritic plaque burden (ITG: *p* = 0.003*; MFG: *p* = 0.002*) and greater neurofibrillary pathology (ITG: *p* < 0.001*; MFG: *p* = 0.010).

#### Polyamine synthesis and catabolism (Fig 6)

**Fig 6:**
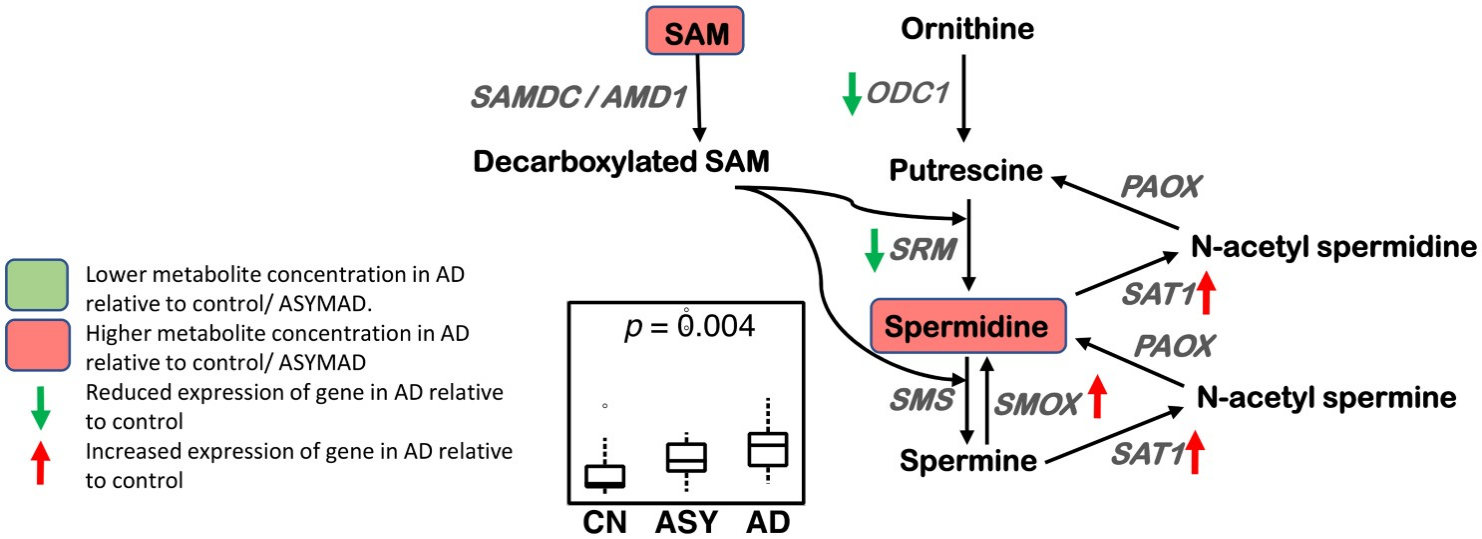
Polyamine synthesis and catabolism. A higher concentration of spermidine is observed in AD in the ITG. The polyamine catabolic genes SAT1 and SMOX have increased expression, and the polyamine synthesis genes SRM and ODC1 have reduced expression in the hippocampus/ERC in AD compared to CN. Sample size: AD = 17, ASY = 13, CN = 13; *p*-values indicate significance after FDR correction for multiple comparisons. AD, Alzheimer disease; ASY, asymptomatic AD; CN, Control; ERC, entorhinal cortex; FDR, false discovery rate; ITG, inferior temporal gyrus; ODC1, ornithine decarboxylase 1; PAOX, peroxisomal N(1)-acetyl-spermine/spermidine oxidase; SAM, S-adenosyl methionine; SAMDC/AMD1, S-adenosylmethionine decarboxylase proenzyme/adenosylmethionine decarboxylase 1; SAT1, spermidine/spermine N1-acetyltransferase 1; SMOX, spermine oxidase; SMS, spermine synthase; SRM, spermidine synthase.

We observed a higher concentration of spermidine (ITG: *p* = 0.004) in AD, as well as associations between higher concentration of spermidine and greater neuritic plaque burden (ITG: *p* = 0.002*; MFG: *p* = 0.013) and greater neurofibrillary pathology (ITG: *p* = 0.003*).

#### Urea cycle (Fig 7)

**Fig 7:**
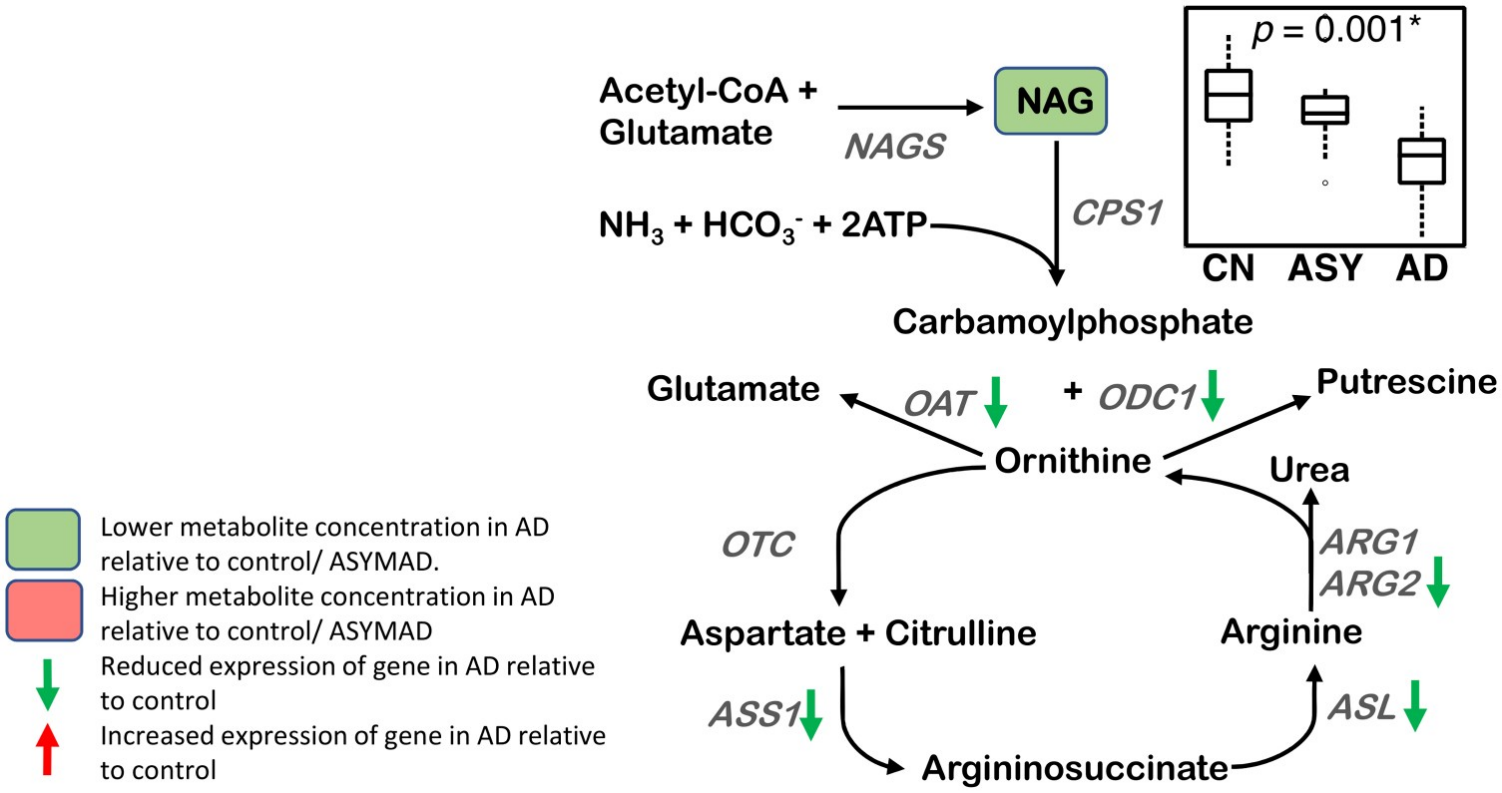
Urea cycle. A lower concentration of NAG is observed in AD in the ITG. The genes ARG2, ASL, ASS1, OAT, and ODC1 have reduced expression in the hippocampus/ERC in AD compared to CN. Sample size: AD = 17, ASY = 13, CN = 13; *p*-values indicate significance after FDR correction for multiple comparisons. An asterisk (*) indicates metabolite was also significant after Bonferroni correction. AD, Alzheimer disease; ARG1, arginase 1; ARG2, arginase 2; ASL, argininosuccinate lyase; ASS, argininosuccinate synthase; ASY, asymptomatic AD; CN, Control; CPS1, carbamoyl phosphate synthetase I; ERC, entorhinal cortex; FDR, false discovery rate; ITG, inferior temporal gyrus; NAGS, N-acetylglutamate synthase; OAT, ornithine aminotransferase; ODC1, ornithine decarboxylase 1; OTC, ornithine transcarbamylase; NAG, N-acetyl glutamate.

We observed a lower concentration of NAG (ITG: *p* < 0.001*; MFG: *p* = 0.031) in AD, as well as associations between lower concentrations of NAG and greater neuritic plaque burden (ITG: *p* = 0.004*) and greater neurofibrillary pathology (ITG: *p* = 0.001*; MFG: *p* = 0.005*).

#### Glutamate-aspartate metabolism (Fig 8)

**Fig 8:**
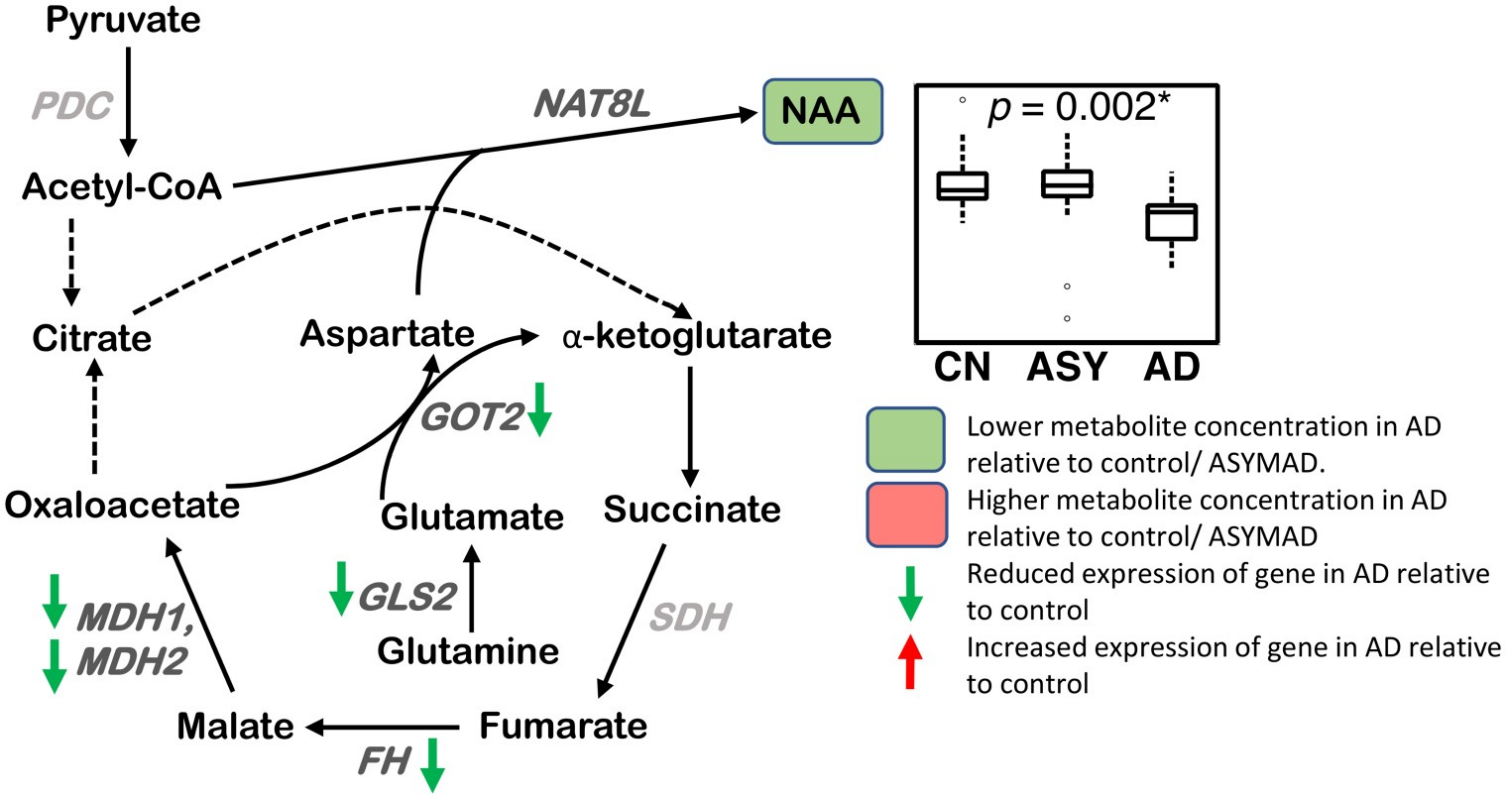
Glutamate-aspartate metabolism. A lower concentration of NAA is observed in AD in the ITG. The genes FH, MDH1, MDH2, GLS2, and GOT2 have reduced expression in the hippocampus/ERC in AD compared to CN. Sample size: AD = 17, ASY = 13, CN = 13; *p*-values indicate significance after FDR correction for multiple comparisons. Genes in light gray indicate expression data were not available. An asterisk (*) indicates metabolite was also significant after Bonferroni correction. Genes in light gray indicate expression data were not available. AD, Alzheimer disease; ASY, asymptomatic AD; CN, Control; CoA, coenzyme A; ERC, entorhinal cortex; FDR, false discovery rate; FH, fumarase; GLS2, glutaminase 2; GOT2, aspartate aminotransferase; ITG, inferior temporal gyrus; MDH1, malate dehydrogenase 1; MDH2, malate dehydrogenase 2; NAA, N-acetyl aspartate; NAT8L, N-acetyltransferase 8 like; PDH, pyruvate dehydrogenase complex; SDH, succinate dehydrogenase.

We observed a lower concentration of NAA (ITG: *p* = 0.002*) in AD, as well as associations between lower concentrations of NAA and greater neuritic plaque burden (ITG: *p* = 0.003*) and greater neurofibrillary pathology (ITG: *p* = 0.005).

#### Neurotransmitter metabolism (Fig 9)

**Fig 9:**
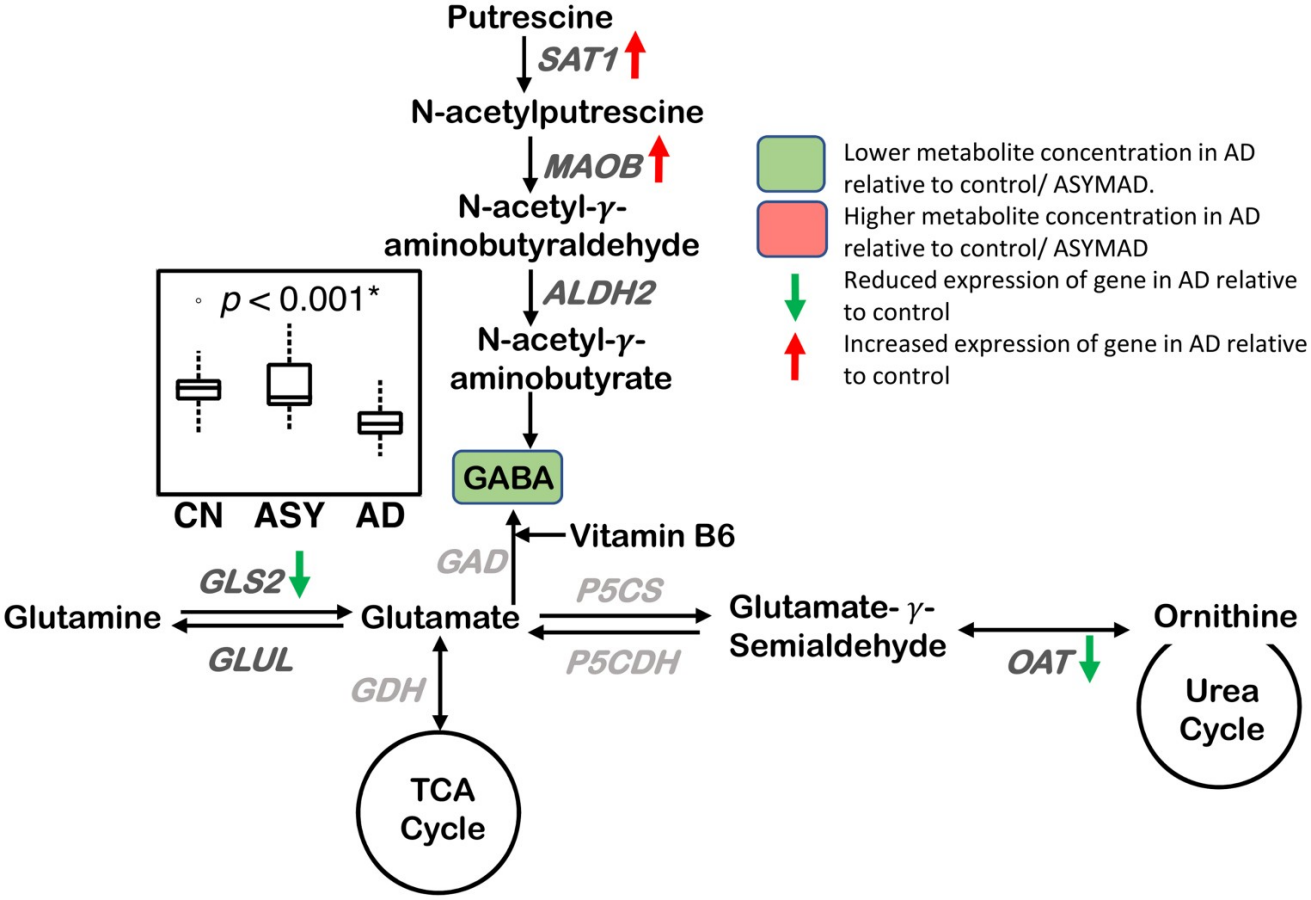
Neurotransmitter metabolism. A lower concentration of GABA is observed in AD in the ITG. The GABA synthesis genes, SAT1 and MAOB, have increased expression, and the genes GLS2 and OAT have reduced expression in the hippocampus/ERC in AD compared to CN. Sample size: AD = 17, ASY = 13, CN = 13; *p*-values indicate significance after FDR correction for multiple comparisons. An asterisk (*) indicates metabolite was also significant after Bonferroni correction. Genes in light gray indicate expression data were not available. AD, Alzheimer disease; ALDH2, aldehyde dehydrogenase 2; ASY, asymptomatic AD; CN, Control; ERC, entorhinal cortex; FDR, false discovery rate; GAD, glutamate decarboxylase; GDH, glutamate dehydrogenase; GLS2, glutaminase 2; GLUL, glutamate-ammonia ligase; ITG, inferior temporal gyrus; MAOB, monoamine oxidase B; OAT, ornithine aminotransferase; P5CDH, delta-1-pyrroline-5-carboxylate dehydrogenase; P5CS, delta-1-pyrroline-5-carboxylate synthase; SAT1, spermidine/spermine N1-acetyltransferase 1/diamine N-acetyltransferase; TCA, tricarboxylic acid cycle.

We observed a lower concentration of GABA (ITG: *p* < 0.001*; MFG: *p* = 0.010) in AD, as well as associations between lower concentrations of GABA and greater neuritic plaque burden (ITG: *p* = 0.003*; MFG: *p* = 0.028) and greater neurofibrillary pathology (ITG: *p* = 0.014; MFG: *p* = 0.007).

For ancillary analyses in the secondary, control brain region (CB), we observed no significant metabolite differences across groups.

### Gene expression

We first tested whether regional gene expression changes in the 71 genes we examined in the inferior temporal and middle frontal cortices were significantly correlated with those in the hippocampus and ERC. We observed that regional gene expression in the ITG was indeed significantly correlated with that in the hippocampus (ρ = 0.455, *p* = 5.9 × 10^−5^) and ERC (ρ = 0.260, *p* = 0.0276), as was the regional gene expression in the MFG (hippocampus: ρ = 0.512, *p* = 4.19 × 10^−6^; ERC: ρ = 0.344, *p* = 0.0031).

**Fig 10:**
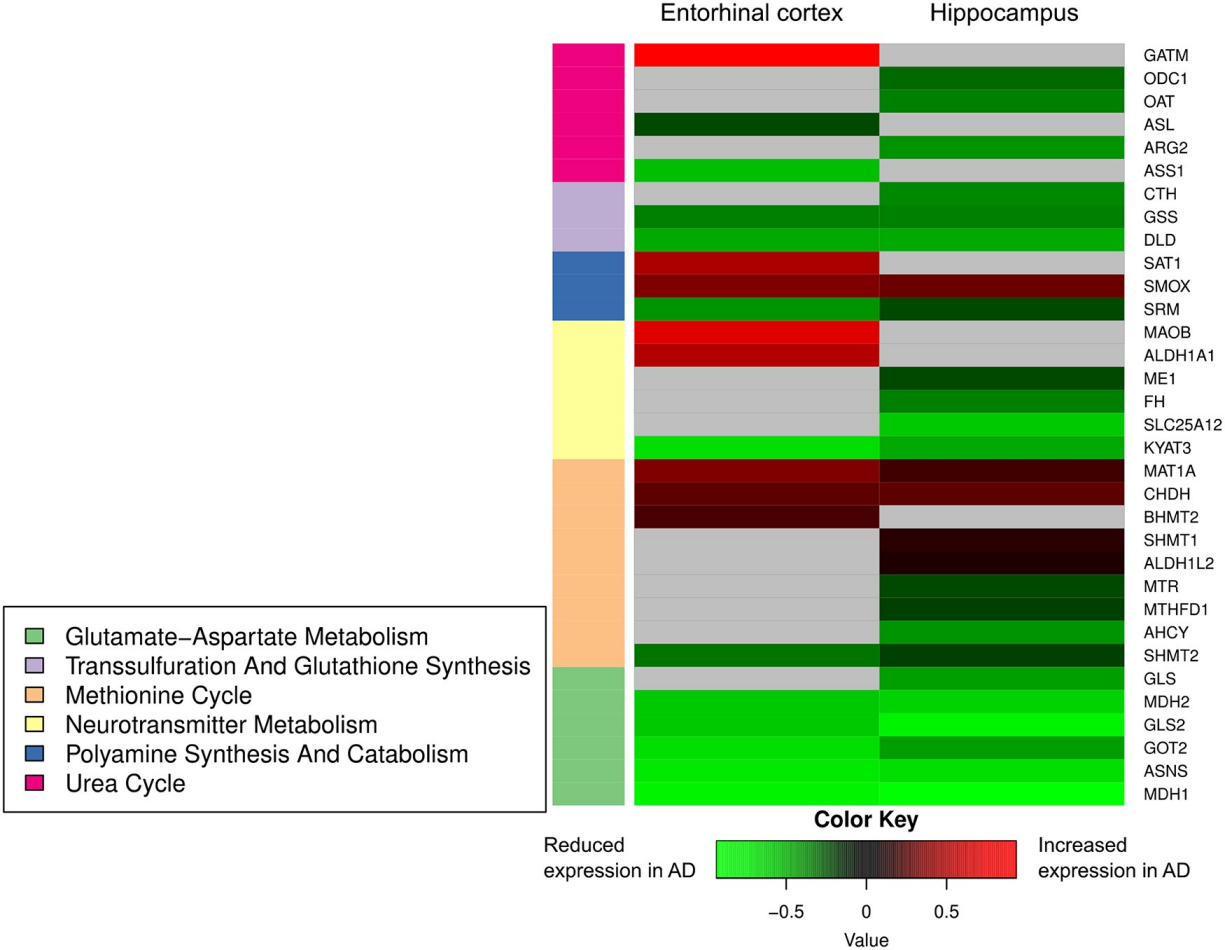
Differential gene expression within ERC and hippocampus. Summary of genes differentially expressed in the hippocampus/ERC in AD compared to controls across all six categories of biochemical reactions related to transmethylation and polyamine metabolism. Gray shading indicates gene expression was not significantly different between AD and control. AD, Alzheimer disease; CN, Control; ERC, entorhinal cortex.

We observed altered levels of gene expression between AD and CN ERC/hippocampus within 33 genes (FDR-corrected *p*-value <0.05) across the six categories representing biochemical reactions related to the transmethylation and polyamine pathways. These differences in gene expression between AD and CN samples were statistically significant after correcting for multiple comparisons using FDR (all results included in Table 3). We present significant results (FDR-corrected *p*-value <0.05) in the heatmap (Fig 10) and additionally visualize significant associations (FDR-corrected *p*-value <0.05) using arrows (with red indicating increased expression in AD and green indicating decreased expression in AD) in each category-specific figure (Figs 4–9). S4 Table lists all the genes whose expression levels differed between AD and CN samples in the ERC/hippocampus together with their known biochemical roles and clinical significance.

**Table 3:** Differences in gene expression between AD and CN participants across category-specific genes.

Gene	ERC			Hippocampus		
	Log fold-change	*p*-value	*p*-value (FDR)	Log fold-change	*p*-value	*p*-value (FDR)
**Methionine Cycle**						
AHCY	−0.186	0.044	0.119	−0.435	<0.001	<0.001**
ALDH1L1	−0.147	0.432	0.584	−0.109	0.521	0.651
ALDH1L2	−0.106	0.048	0.126	0.111	0.012	0.040*
AMD1	0.064	0.52	0.661	−0.048	0.623	0.736
BHMT	−0.008	0.943	0.967	−0.128	0.241	0.376
BHMT2	0.244	0.012	0.049*	0.155	0.123	0.228
CHDH	0.298	0.002	0.012*	0.286	<0.001	0.005**
DMGDH	−0.017	0.791	0.87	−0.065	0.299	0.439
MAT1A	0.351	<0.001	<0.001**	0.224	<0.001	0.005**
MTHFD1	−0.177	0.049	0.128	−0.225	0.013	0.044*
MTHFD2	0.484	0.111	0.228	−0.042	0.852	0.903
MTHFR	0.046	0.425	0.578	0.116	0.016	0.051
MTR	−0.16	0.185	0.327	−0.23	0.01	0.034*
MTRR	0.18	0.119	0.239	−0.002	0.986	0.992
SARDH	0	0.996	0.997	0.066	0.305	0.445
TYMS	0.389	0.041	0.114	0.043	0.819	0.882
SHMT1	0.172	0.052	0.134	0.174	0.007	0.027*
SHMT2	−0.336	<0.001	0.005**	−0.209	0.004	0.019*
**Transsulfuration and Glutathione Synthesis**						
AMT	0.181	0.019	0.067	0.052	0.501	0.635
CTH	−0.115	0.174	0.313	−0.402	<0.001	0.001**
DLD	−0.491	0.002	0.016*	−0.514	<0.001	<0.001**
GCLC	−0.205	0.07	0.165	−0.224	0.032	0.083
GCLM	0.037	0.693	0.8	−0.064	0.33	0.472
GLDC	0.152	0.13	0.255	−0.231	0.031	0.08
GSS	−0.363	<0.001	<0.001**	−0.349	<0.001	<0.001**
**Polyamine Synthesis and Catabolism**						
PAOX	−0.177	0.027	0.086	−0.113	0.147	0.26
SAT1	0.478	<0.001	0.007**	0.184	0.102	0.198
SAT2	−0.07	0.396	0.551	−0.063	0.374	0.515
SMOX	0.36	<0.001	0.003**	0.301	<0.001	0.001**
SRM	−0.418	<0.001	0.001**	−0.226	0.006	0.024*
**Urea Cycle**						
ARG1	−0.051	0.549	0.685	0.025	0.72	0.81
ARG2	−0.261	0.022	0.073	−0.416	0.002	0.010**
ASL	−0.239	0.008	0.036*	−0.079	0.316	0.457
ASS1	−0.556	0.002	0.014*	−0.233	0.082	0.168
CPS1	0.093	0.256	0.407	0.02	0.803	0.871
GATM	0.936	<0.001	<0.001**	0.029	0.792	0.862
NAGS	0.132	0.142	0.271	0.187	0.018	0.054
OAT	−0.172	0.331	0.488	−0.369	<0.001	0.002**
ODC1	−0.071	0.603	0.729	−0.3	0.014	0.044*
OTC	0.146	0.126	0.249	0.172	0.025	0.07
PYCR1	0.019	0.837	0.898	0.131	0.046	0.109
PYCR2	0.083	0.266	0.418	−0.046	0.509	0.641
**Glutamate-Aspartate Metabolism**						
ASNS	−0.752	<0.001	<0.001**	−0.722	<0.001	<0.001**
GLS	−0.344	0.02	0.068	−0.467	<0.001	<0.001**
GLS2	−0.624	0.003	0.019*	−0.904	<0.001	<0.001**
GLUL	0.285	0.059	0.146	−0.007	0.955	0.971
GOT2	−0.713	<0.001	0.001**	−0.444	<0.001	<0.001**
MDH1	−0.888	<0.001	0.009**	−0.929	<0.001	<0.001**
MDH2	−0.596	<0.001	0.004**	−0.673	<0.001	<0.001**
PDC	0.061	0.331	0.487	−0.031	0.539	0.667
**Neurotransmitter Metabolism**						
ALDH1A1	0.537	0.006	0.030*	−0.401	0.099	0.194
ALDH2	0.134	0.126	0.248	0.034	0.683	0.782
COMT	0.009	0.919	0.953	0.111	0.05	0.117
DBH	−0.133	0.057	0.143	0.133	0.061	0.135
DDC	0.012	0.807	0.88	0.043	0.488	0.623
FAH	−0.032	0.804	0.878	−0.089	0.433	0.572
FH	−0.197	0.024	0.077	−0.372	<0.001	<0.001**
GSTZ1	0.119	0.159	0.294	0.033	0.732	0.819
HGD	0.055	0.324	0.48	0.11	0.019	0.058
HPD	0.053	0.461	0.61	0.092	0.639	0.748
KYAT1	−0.023	0.705	0.81	0.033	0.612	0.727
KYAT3	−0.72	<0.001	<0.001**	−0.484	<0.001	0.006**
MAOA	0.039	0.723	0.823	0.1	0.336	0.478
MAOB	0.726	<0.001	<0.001**	0.172	0.16	0.278
ME1	−0.159	0.213	0.36	−0.238	0.01	0.036*
NAT8L	−0.069	0.368	0.524	−0.072	0.366	0.508
PAH	0.072	0.687	0.796	0.022	0.87	0.916
PNMT	−0.029	0.775	0.858	0.12	0.098	0.193
SLC25A12	−0.431	0.02	0.069	−0.592	<0.001	<0.001**
TAT	−0.04	0.5	0.643	−0.026	0.475	0.611
TH	−0.2	0.284	0.439	−0.279	0.088	0.178

*Indicates FDR-corrected *p*-value <0.05.

**Indicates FDR-corrected *p*-value <0.01.

Abbreviations: AD, Alzheimer disease; CN, control; ERC, entorhinal cortex; FDR, false discovery rate

## Discussion

In this study, we extended our prior work demonstrating abnormal glycolysis [8] and fatty acid [10] and phospholipid metabolism [9] in AD. We examined whether dysregulation in the transmethylation pathway, the primary metabolic source of one-carbon units, and the closely related polyamine synthesis/catabolism pathway were associated with AD pathogenesis. Our findings provide evidence for broad dysregulation of transmethylation and polyamine synthesis/catabolism, including abnormalities in neurotransmitter signaling, urea cycle, aspartate-glutamate metabolism, and glutathione synthesis.

### Methionine cycle (Fig 4)

We observed lower choline concentrations across all three brain regions in AD, as well as associations with more severe amyloid pathology and neurofibrillary pathology. To the best of our knowledge, this is the first study to measure absolute concentrations of free choline in AD brain tissue samples and relate lower levels to greater severity of AD pathology. Using an untargeted metabolomics approach, Paglia and colleagues reported increased levels of choline in AD in the frontal cortex [46]. Methodological differences (quantitative targeted versus untargeted metabolomics) may account for this inconsistent finding [46]. Most previous studies examining brain choline concentrations in AD have used proton magnetic resonance spectroscopy (^1^H-MRS); that detects the choline compounds, glycerophosphocholine, and phosphocholine. These studies, as well as those measuring ratios of choline to creatine, have been largely inconsistent [47–51]. Our current findings are significant as they suggest that decreased brain choline concentrations may represent a shift in choline metabolism in the AD brain through enhanced flux in the methionine cycle and transmethylation reactions. Adding further support to this hypothesis is our observation of overexpression of the choline dehydrogenase (CHDH) gene in both the ERC and hippocampus in AD. This flavin-dependent mitochondrial enzyme oxidizes choline to betaine aldehyde in the methionine cycle [52]. Together with our recent findings of altered phosphatidylcholine metabolism in AD [9], these results suggest that enhanced utilization of choline in membrane remodeling by incorporation into phospholipids, as well as in the transmethylation pathway by its conversion to betaine, may reduce its availability for the synthesis of acetylcholine within basal forebrain cholinergic neurons. This hypothesis merits further confirmation in experimental studies.

The role of choline in the methionine cycle (Fig 4) is through its oxidation to betaine by CHDH and the subsequent transfer of a methyl group from betaine to homocysteine to generate methionine in a reaction catalyzed by betaine-homocysteine methyltransferase (BHMT). Alternatively, another homocysteine methyltransferase enzyme called 5-methyltetrahydrofolate-homocysteine methyltransferase (MTR) uses 5-methyltetrahydrofolate (5-MTHF) as a methyl donor to convert homocysteine to methionine. The subsequent conversion of methionine to SAM occurs in a two-step reaction catalyzed by methionine adenosyltransferase 1A (MAT1A). SAM plays a critical role as a universal methyl group donor, participating in more than 200 enzymatic reactions including phospholipid, DNA, RNA, and protein methylation [53]. It is therefore striking that we find a significantly higher concentration of SAM in the ITG in AD compared to controls; higher SAM concentration was also associated with more severe neuritic plaque and neurofibrillary tangle pathology. Consistent with our results, Paglia and colleageus observed an increase in SAM concentrations in the frontal cortex in AD [46]. However, in contrast to these results, a previous postmortem study in humans found decreased SAM in all cortical regions examined compared to controls [4]. Both methodological differences in measuring SAM concentrations as well as differences in the participant’s age (75–76 years versus 86 years) and duration of disease may account for this inconsistency.

Our transcriptomic analysis in the hippocampus and ERC provides complementary evidence for alterations in the genetic regulators of enzymatic reactions of the methionine cycle in AD. Besides altered gene expression of CHDH, we found differential gene expression of the two homocysteine methyltransferase enzymes, BHMT2 and MTR, that catalyze the conversion of homocysteine to methionine. We also observed lower expression of the MTHFD1 gene that encodes a trifunctional C1-tetrahydrofolate (THF) synthetase catalyzing the ATP-dependent conversion of THF and formate to 5,10-MethyleneTHF (CH_2_THF). The irreversible reduction of CH_2_THF by the enzyme 5,10-methylene THF reductase (MTHFR) generates 5-methylTHF, a key methyl donor for the remethylation of homocysteine to methionine [54]. These findings add to emerging evidence for methionine metabolism as an important modulator of neurodegeneration as well as a metabolic determinant of longevity in several animal models [55–57]. Intriguingly, recent genome-wide association studies (GWAS) have implicated polymorphic variations in MAT1A as being associated with both longevity and cognitive performance during aging [58]. Our findings are also consistent with a previous study implicating elevated plasma homocysteine levels as risk factors for both cerebrovascular disease and AD [54,59].

Strikingly, we also found overexpression of serine hydroxymethyltransferase 1 (SHMT1) in the hippocampus in AD. SHMT1 is a pyridoxal phosphate-containing enzyme that catalyzes the reversible conversion of serine and THF to glycine and CH_2_THF. Besides its role as a methyl group donor for the remethylation of homocysteine, CH_2_THF is also a source of methyl groups for the synthesis of thymidylate or deoxythimidine monophosphate (dTMP) from deoxyuridine monophosphate (dUMP). The net effect of reduced CH_2_THF may be impaired de novo dTMP synthesis and a greater rate of uracil misincorporation into DNA, resulting in single- and double-stranded breaks through the actions of DNA repair enzymes [60]. Our findings assume importance given previous studies implicating genome instability and impaired DNA repair in AD pathogenesis [61].

### Transsulfuration and glutathione synthesis (Fig 5)

Besides its role in the methionine cycle as a substrate for conversion to methionine, an alternative metabolic fate of homocysteine is in the transsulfuration pathway (Fig 5), where it can be used to generate cysteine, a precursor of the principal antioxidant tripeptide, glutathione. Our metabolomics analyses revealed significantly higher concentrations of both cysteine and thiol-reduced glutathione (GSH) in the ITG in AD as well as associations with both metabolites and more severe amyloid and neurofibrillary pathology. These results suggest that AD is characterized by a state of increased oxidative stress with a greater demand for recruitment of antioxidant defense mechanisms through GSH. Additionally, besides its well-established role as a free radical scavenger, GSH is also involved in detoxification of electrophiles and plays numerous roles in diverse cellular functions of relevance to neurodegeneration, including DNA synthesis, posttranslational modification of proteins and immune function [62,63]. Our results suggest that higher levels of cysteine, the rate-limiting substrate for GSH synthesis, are indicative of a greater drive towards generation of GSH in brain regions vulnerable to AD pathology. These findings are consistent with a previous postmortem metabolomics analysis in humans by Xu and colleagues that showed significantly higher levels of cysteine in the hippocampus and cingulate gyrus in AD [1]. Previous MRS studies have shown inconsistent findings with reports of decreased GSH levels in the frontal cortex and hippocampus in AD, whereas MCI individuals appear to have increased GSH levels in the anterior and posterior cingulate cortices. The latter report by Duffy and colleagues is especially relevant in the context of our current findings, as the authors hypothesize that higher GSH levels in early stages of AD progression may be a compensatory mechanism to counter cysteine-induced excitotoxicity through activation of N-methyl-D-aspartate (NMDA) receptors [64].

Consistent with our metabolomics results, we found altered expression of genes encoding enzymes catalyzing reactions essential for GSH synthesis in the hippocampus/ERC in AD. These include significantly reduced expression of cystathionine gamma-lyase (CTH), which breaks down cystathionine into cysteine, the rate-limiting substrate for glutathione synthesis as well as GSH synthase (GSS), catalyzing the second step in GSH synthesis.

### Polyamine synthesis and catabolism (Fig 6)

We observed a significantly higher concentration of spermidine in the ITG in AD, as well as associations with more severe amyloid pathology and neurofibrillary pathology. Our results are broadly consistent with other studies on human postmortem samples comparing AD and control brains as well as a mouse model of AD showing increased brain polyamine concentrations [2,3,65]. Ornithine decarboxylase 1 (ODC1) is the first rate-limiting enzyme in polyamine biosynthesis (Fig 6) and catalyzes the conversion of ornithine, generated in the urea cycle (discussed below) to putrescine. The generation of SAM in the methionine cycle (described above) is involved in the second rate-limiting step of spermidine and spermine biosynthesis through its decarboxylation by the enzyme SAM decarboxylase (AMD1) [66]. This reaction generates decarboxylated SAM that is a source of aminopropyl groups, which are successively added to putrescine to generate spermidine and spermine in reactions catalyzed by spermidine synthase (SRM) and spermine synthase (SMS), respectively. Taken together with our observation of increased SAM concentration in AD, higher concentrations of spermidine may suggest enhanced drive towards greater polyamine biosynthesis in AD. Altered brain polyamine metabolism may impact AD progression through several mechanisms, including modulation of cholinergic neurotransmission and activity of NMDA receptors [67]. While high polyamine concentrations have been shown to reduce neuronal survival, they provide trophic support during synaptogenesis in the developing brain and are therefore likely to play complex, pleiotropic roles in AD progression [68–70].

Our transcriptomic analysis suggests differential regulation of both polyamine biosynthesis and catabolism in AD with reduced expression of SRM and ODC1 and increased expression of the catabolic enzymes spermine oxidase (SMOX) and spermine/spermidineN1-acetyltransferase (SAT1). ODC1 encodes ornithine decarboxylase, the rate-limiting enzyme in the biosynthesis of polyamines, while SAT1 is the rate-limiting enzyme of polyamine catabolism and catalyzes the acetylation of spermine and spermidine, which can then be oxidized by polyamine oxidase (PAOX). The role of SAT1 in mental illness has been particularly well studied in major depression and suicide and is one of the most consistently implicated genes in these conditions [71,72]. The degradation of polyamines by SMOX and PAOX results in the generation of hydrogen peroxide and the neurotoxic aldehyde 3-aminopropanal (3-AP) [73,74]. Reactive aldehyde species such as 3-AP have been extensively studied as cytotoxic metabolites in neurodegeneration as well as cerebral ischemia [75]. Together, our findings suggest both direct cytotoxic effects from increased polyamine synthesis as well as from reactive aldehydes generated through enhanced polyamine catabolism in AD.

### Urea cycle (Fig 7)

We observed lower concentrations of N-acetyl glutamate (NAG) in the ITG in AD, as well as associations with lower NAG concentrations and more severe amyloid and neurofibrillary pathology. These results are similar to those reported by Xu et al. who observed lower NAG levels in the ERC in AD using GC-MS. However, in contrast to prior reports by Xu et al. and Liu et al. using LC-MS, we did not find lower brain concentration of ornithine in AD [1,2]. Abnormalities in the urea cycle and their potential role in AD have received attention after microarray experiments showed altered expression of the urea cycles enzymes in the AD brain [76]. The primary regulated step in the urea cycle is the reaction catalyzed by the mitochondrial enzyme, carbamoyl phosphate synthetase 1 (CPS1), that synthesizes carbamoyl phosphate from inorganic ammonium and carbonate (Fig 7). CPS1 requires NAG as an essential cofactor, which is synthesized in a reaction catalyzed by another mitochondrial enzyme, N-acetylglutamate synthase (NAGS) from glutamate and acetyl CoA. We observed differences in gene expression of key regulators of the urea cycle; in particular, the mitochondrial enzymes ASS1, OAT, ARG2, ODC1, and the cytosolic enzyme ASL showed reduced expression in the ERC/hippocampus in AD. ASS1 catalyzes the conversion of citrulline to argininosuccinate, the rate-limiting step in arginine synthesis, and together with ASL, recycles citrulline into arginine in the urea cycle. OAT catalyzes the conversion of ornithine into the major excitatory and inhibitory neurotransmitters glutamate and GABA, respectively [77]. In agreement with previous studies that showed altered brain arginase activity in AD [2], we found reduced expression of ARG2. Previous gene expression analyses have reported both increased and reduced expression of ARG2 in the AD brain [76,78]. Taken together, our findings add further evidence implicating dysregulation of the urea cycle as a key metabolic abnormality in AD that can also impact other biochemical pathways including polyamine metabolism (as discussed above) as well as neurotransmitter metabolism (discussed below).

### Glutamate-aspartate metabolism (Fig 8)

Consistent with numerous prior studies using ^1^H-MRS that have shown lower levels of NAA in AD within regions vulnerable to pathology, such as the posterior cingulate cortex and hippocampus [51], our CE-MS assays also showed evidence of lower NAA concentration in the ITG in AD as well as associations with lower concentrations and more severe amyloid pathology and neurofibrillary pathology. Besides being a well-recognized neuroimaging marker of neuronal metabolic integrity, NAA plays several important biological roles including in myelin turnover, mitochondrial energy production, neuronal osmoregulation, and neuron-glia signaling [79–81]. Its synthesis within neurons occurs in an energy-dependent reaction that is catalyzed by neuron-specific N-acetyltransferase (NAT8L) (Fig 8), utilizing glutamate as a transamination source for aspartate and either pyruvate or 3-hydroxybutyrate as a source of acetyl CoA [80]. While lower NAA levels in neurodegenerative diseases may be a marker of neuronal loss, they may also reflect compromised NAA synthesis within neurons facing increasing bioenergetic demand due to perturbations in mitochondrial oxidative phosphorylation. In this context, it is interesting to note that Zaroff and colleagues recently reported under-expression of *NAT8L* as well as NAA levels in a transgenic mouse model of AD, even in the absence of significant neuronal loss [81]. Our findings are in broad agreement with those of Paglia and colleagues who showed evidence for dysregulation in brain aspartate and glutamate metabolism in AD [46]. There are significant methodological differences between this prior report and our current study, in that Paglia and colleagues used an untargeted metabolomics approach. This may explain some inconsistencies between our current results and their findings, including their observation of increased NAA levels in AD.

Our transcriptomic results add further support to evidence that mitochondrial energy production is a key abnormality in AD. We observed significantly reduced expression of the glutamate oxaloacetate transaminase (GOT2) gene in both the ERC and hippocampus in AD. GOT2 catalyzes a key mitochondrial transamination reaction that allows rapid generation of ATP by bypassing initial steps in the TCA cycle. This reaction is essential to the “mini citric acid cycle” described by Yudkoff and colleagues and allows rapid energy generation required for neuronal activity [82]. Moreover, aspartate generated in this reaction can serve as a substrate for NAA production. Therefore, our findings of lower NAA concentration and reduced expression of GOT2 further implicate impaired mitochondrial energy production in AD pathogenesis.

### Neurotransmitter metabolism (Fig 9)

Our observation of lower GABA concentration in both the ITG and MFG in AD is consistent with prior studies in postmortem brain tissue samples [51,83–85]. Additionally, we found that lower GABA concentration across all three brain regions was associated with more severe amyloid pathology and neurofibrillary pathology. This suggests that impaired GABA neurotransmission may be a regionally widespread phenomenon that may impact severity of AD pathology. These findings are especially relevant, as impaired balance between excitatory and inhibitory neurotransmission is emerging as an important mediator of clinical progression in AD, and modulation of GABA signaling may be a novel therapeutic strategy for disease modification [86]. Our gene expression analyses also revealed overexpression of MAO-B, a gene regulating neurotransmitter catabolism, in the ERC. While these results are consistent with early autoradiographic studies that revealed significantly higher MAO-B activity in the AD brain [87], increased MAO-B uptake detected by positron emission tomography (PET) is emerging as a promising biomarker of neuroinflammation and reactive astrocytosis in early stages of AD [88]. A direct role for MAO-B in AD is also suggested by recent findings that that MAO-B is a γ-secretase–associated protein capable of regulating intraneuronal Aβ levels [89]. MAO-B also catalyzes the second of four sequential reactions in the synthesis of GABA from putrescine, by converting N-acetylputrescine to N-acetyl-γ-aminobutyraldehyde. This reaction is known to occur predominantly within astrocytes [90,91]. Thus, our results suggest that the up-regulation of MAO-B may be a compensatory mechanism against presumably lower GABA biosynthesis within neuronal cells [92].

**Fig 11:**
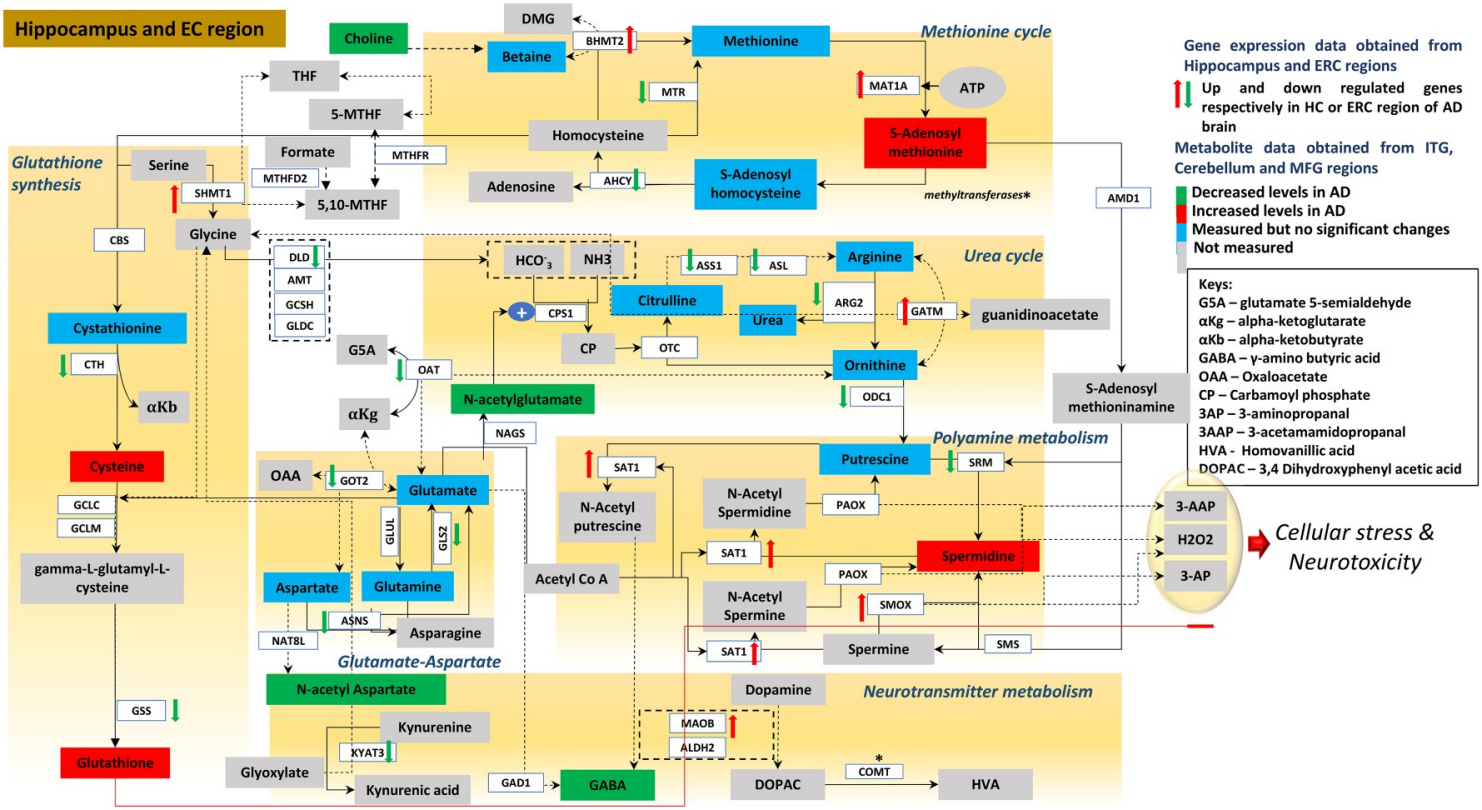
Integrated summary of alterations in metabolite concentrations and gene expression within metabolic pathways linked to transmethylation and polyamine synthesis/catabolism. Reactions within each of the six categories related to transmethylation and polyamine metabolism are shown within brown boxes [93–95]. Metabolites whose concentrations are increased in the AD ITG relative to controls are shown in red boxes; those that are reduced in AD are shown in green boxes. Metabolites whose concentrations were measured but did not differ between AD and control are indicated in blue boxes; metabolites whose concentrations were not assayed are shown in gray boxes. Red up and green down arrows indicate significantly increased and reduced gene expression in the ERC/hippocampus, respectively. AD, Alzheimer disease; AHCY, adenosylhomocysteinase; ALDH2, aldehyde dehydrogenase 2; AMD1, adenosylmethionine decarboxylase 1; AMT, aminomethyltransferase; ARG2, arginase 2; ASL, argininosuccinate lyase; ASNS, asparagine synthetase; ASS1, argininosuccinate synthase 1; BHMT2, betaine homocysteine s-methyltransferase 2; CBS, cystathionine beta synthase; CN, Control; COMT, catechol-o-methyltransferase; CPS1, carbamoyl phosphate synthase 1; CTH, cystathionine gamma-lyase; DLD, dihydrolipoamide dehydrogenase; ERC, entorhinal cortex; GAD1, glutamate decarboxylase 1; GATM, glycine amidino transferase; GCLC, glutamate cysteine ligase catalytic; GCLM, glutamate cysteine ligase modifier; GCSH, glycine cleavage system protein H; GLDC, glycine decarboxylase; GLS2, glutaminase 2; GLUL, glutamine synthetase; GOT2, glutamic oxaloacetic transaminase 2; GSS, GSH synthetase; HC, hippocampus; ITG, inferior temporal gyrus; KYAT3, kynurenine aminotransferase 3; MAT1A, methionine adenosyltransferase 1A; MFG, middle frontal gyrus; MOAB, monoamine oxidase B; MTHFD2, methylenetetrahydrofolate dehydrogenase 2; MTHFR, methylenetetrahydrofolate reductase; MTR, methyltransferase; NAT8L, n-acetyltransferase 8 like; OAT, ornithine aminotransferase; ODC1, ornithine decarboxylase 1; OTC, ornithine carbamoyltransferase; PAOX, polyamine oxidase; SAT1, spermidine/spermine n1-acetyltransferase 1; SHMT1, serine hydroxymethyl transferase 1; SMOX, spermine oxidase; SMS, spermine synthase; SRM, spermidine synthase.

Fig 11 summarizes our overall findings by integrating metabolite and gene expression results across all six categories of biochemical reactions we examined and clearly shows how these pathways intersect and potentially interact at numerous levels to influence severity of AD pathology and symptom expression.

### Conclusions and limitations

To the best of our knowledge, this is the first study to apply targeted metabolomics using CE-MS in brain tissue samples in combination with analyses of gene expression to identify abnormalities in multiple metabolic networks related to the transmethylation and polyamine pathways in AD. Some limitations of our study merit consideration. Our CE-MS–based metabolomics assays detected only a small proportion of all metabolites participating in the pathways we examined. Our interpretation of the results is therefore limited to analytes that could be reliably detected. The analyses of gene expression were performed on previously published and publicly available datasets distinct from the BLSA. We overlaid gene expression results from these datasets on our metabolomics analyses to enhance interpretability of our findings and derive plausible biological mechanisms underlying changes in brain metabolite levels in AD. Furthermore, we focused on gene expression profiles in the ERC and hippocampus, whereas our metabolomics assays were performed in the ITG, MFG, and CB. However, we confirmed that gene expression changes in the ITG were correlated with those in the ERC and hippocampus. Our rationale to focus on the ERC and hippocampus was that accumulation of pathology in these regions is thought to trigger onset of cognitive impairment in AD [27–29]. The limited availability of tissue samples from the ERC and hippocampus precluded metabolomics assays on these two regions in our study. Another limitation was the cross-sectional nature of our metabolomic and transcriptomic analyses. We were therefore unable to directly assess how AD progression may impact changes in brain tissue concentrations of metabolites or up- or down-regulation of genes within specific pathways. This study design also makes any definitive conclusions about directionality of metabolite and gene expression changes challenging. Thus, we are unable to assess whether up- or down-regulation of gene expression lead to changes in metabolite concentrations or represent adaptive responses to alterations in metabolite levels. However, integrating metabolomics and transcriptomics analyses to map a priori–specified biochemical pathways may provide important insights into how AD may broadly impact multiple interlinked metabolic pathways. An additional limitation is that our brain tissue and gene expression sample size was small, and we explored associations across multiple metabolites and genes within a limited sample. We were therefore likely underpowered to detect all alterations in the pathway-specific metabolites and genes that we studied. Additionally, studies with small sample sizes may be underpowered to extrapolate results to a larger population [96], and significant results may not reflect true effects [97]. Considering these sample size limitations, we specified a priori hypotheses with metabolites of interest and implemented FDR and Bonferroni adjustments to control for multiple comparisons to reduce potential biases. Finally, it is also worth considering that in an autopsy-based assessment of a neurodegenerative disease such as AD, some observed changes may be nonspecific markers of terminal events related to neuronal loss. The specificity of our findings merit testing in other neurodegenerative diseases in future studies.

Our results implicate alterations in cellular methylation potential and increased flux in the transmethylation pathways, increased demand on antioxidant defense mechanisms, perturbations in intermediate metabolism in the urea cycle and aspartate-glutamate pathways disrupting mitochondrial bioenergetics, increased polyamine biosynthesis and breakdown, as well as abnormalities in neurotransmitter metabolism that are related to severity of AD pathology and the expression of clinical symptoms. They illustrate the contours of AD as a pervasive metabolic disorder that may involve dysregulation of multiple interconnected metabolic pathways that are each capable of triggering accumulation of AD pathology and eventual evolution of symptoms. We believe that this study significantly adds to a comprehensive understanding of the metabolic basis of AD pathogenesis and provides insights into novel targets for disease-modifying therapies.

## Supporting information

S1 STROBE Checklist (DOCX)

S1 Table Metabolites studied in this report, including their class, ion type, and *m/z* and MT data. *Indicates that relative measurements were performed for these two metabolites. The values provided represent the average areas of urea and NAA peaks relative to the methionine sulfone internal standard added to the test samples. **Spermine was dropped from the analysis due to above threshold (>30%) missing values. Count/120 denotes the total number of samples in which the metabolite was detected. *m/z*, mass-to-charge ratio; MT, migration time; NAA, N-acetylaspartate; S/N; signal-to-noise ratio.
(DOCX)

S2 Table Regional brain tissue concentrations (picomoles per milligram of brain tissue) of metabolites assayed by CE-TOFMS. *Indicates metabolites that could only be relatively quantified. The values provided represent the average areas of urea and NAA peaks relative to the methionine sulfone internal standard added to the test samples. AD, Alzheimer disease; ASY, asymptomatic AD; CB, cerebellum; CE-TOFMS, capillary electrophoresis time-of-flight mass spectrometry; CN, control; ITG, inferior temporal gyrus; MFG, medial frontal gyrus; NAA, N-acetylaspartate.
(DOCX)

S3 Table Group differences in the CB. *p*-Value indicates significance after FDR correction for 26 comparisons. Group differences were tested using linear mixed-effects models for each metabolite category. Bon, Bonferroni; CB, cerebellum, FDR, false discovery rate.
(DOCX)

S4 Table Role and clinical significance of genes differentially expressed in AD compared to CN. ^a^Compiled from open source references, including GeneCards (genecards.org) and Wikipedia (wikipedia.org). Note: gene expression significance was calculated in the hippocampus and ERC regions; genes represented in the table were significantly different between AD and CN individuals in at least one of the two regions. AD, Alzheimer disease; CN, control; ERC, entorhinal cortex.
(DOCX)

## Abbreviations

^1^H-MRS: proton magnetic resonance spectroscopy
3-AP: 3-aminopropanal
5-MTHF: 5-methyltetrahydrofolate
AD: Alzheimer disease
AMD1: *S*-adenosylmethionine decarboxylase
*APOE* ε4: apolipoprotein *E*
ASY: asymptomatic AD
Aβ: amyloid beta
BHMT: betaine-homocysteine methyltransferase
BLSA: Baltimore Longitudinal Study of Aging
CB: cerebellum
CE-MS: capillary electrophoresis-mass spectrometry
CERAD: Consortium to Establish a Registry for Alzheimer’s Disease
CE-TOFMS: capillary electrophoresis time-of-flight mass spectrometry
CH_2_THF: 5,10-MethyleneTHF
CHDH: choline dehydrogenase
CN: Control
CoA: coenzyme A
CTH: cystathionine gamma-lyase
CV: coefficient of variation
dTMP: deoxythimidine monophosphate
dUMP: deoxyuridine monophosphate
ERC: entorhinal cortex
FDR: false discovery rate
FIA-MS/MS: flow injection analysis-mass spectrometry
FWER: family-wise error rate
GEE: generalized estimating equation
GEO: Gene Expression Omnibus
GOT2: glutamate oxaloacetate transaminase
GSH: reduced glutathione
GSS: GSH synthase
GSSG: glutathione disulfide/oxidized glutathione
GWAS: genome-wide association study
ITG: inferior temporal gyrus
LC-MS/MS: liquid chromatography-tandem mass spectrometry
*m/z*: mass-to-charge ratio
MAT1A: methionine adenosyltransferase 1A
MCI: mild cognitive impairment
MFG: middle frontal gyrus
MT: migration time
MTHFR: 5,10-methylene tetrahydrofolate reductase
MTR: 5-methyltetrahydrofolate-homocysteine methyltransferase
NAA: N-acetylaspartate
NAG: N-acetylglutamate
NAGS: N-acetylglutamate synthase
NAT8L: N-acetyltransferase
NIA: National Institute on Aging
NMDA: N-methyl-D-aspartate
OCM: one-carbon metabolism
ODC: ornithine decarboxylase
PAOX: polyamine oxidase
PET: positron emission tomography
PMI: postmortem interval
RMA: Robust Multi-array Average
S/N: signal-to-noise ratio
SAH: S-adenosylhomocysteine
SAM: S-adenosylmethionine
SAT1: spermine/spermidineN1-acetyltransferase
SDMA: symmetric dimethylarginine
SHMT1: serine hydroxymethyltransferase
SMOX: spermine oxidase
SMS: spermine synthase
SRM: spermidine synthase
THF: tetrahydrofolate

## References

[1] Xu J, Begley P, Church SJ, Patassini S, Hollywood KA, Jullig M, et al Graded perturbations of metabolism in multiple regions of human brain in Alzheimer’s disease: Snapshot of a pervasive metabolic disorder. Biochim Biophys Acta. 2016;1862(6):1084–92. Epub 2016/03/10. 10.1016/j.bbadis.2016.03.001 26957286 PMC4856736

[2] Liu P, Fleete MS, Jing Y, Collie ND, Curtis MA, Waldvogel HJ, et al Altered arginine metabolism in Alzheimer’s disease brains. Neurobiol Aging. 2014;35(9):1992–2003. Epub 2014/04/22. 10.1016/j.neurobiolaging.2014.03.013 24746363

[3] Inoue K, Tsutsui H, Akatsu H, Hashizume Y, Matsukawa N, Yamamoto T, et al Metabolic profiling of Alzheimer’s disease brains. Sci Rep. 2013;3:2364 Epub 2013/08/07. 10.1038/srep02364 23917584 PMC3734482

[4] Morrison LD, Smith DD, Kish SJ. Brain S-adenosylmethionine levels are severely decreased in Alzheimer’s disease. J Neurochem. 1996;67(3):1328–31. Epub 1996/09/01. 10.1046/j.1471-4159.1996.67031328.x 8752143

[5] Cedernaes J, Schioth HB, Benedict C. Efficacy of antibody-based therapies to treat Alzheimer’s disease: just a matter of timing? Exp Gerontol. 2014;57:104–6. Epub 2014/05/20. 10.1016/j.exger.2014.05.002 24835192

[6] Rosenblum WI. Why Alzheimer trials fail: removing soluble oligomeric beta amyloid is essential, inconsistent, and difficult. Neurobiol Aging. 2014;35(5):969–74. Epub 2013/11/12. 10.1016/j.neurobiolaging.2013.10.085 24210593

[7] Ganjei JK. Targeting amyloid precursor protein secretases: Alzheimer’s disease and beyond. Drug News Perspect. 2010;23(9):573–84. Epub 2010/12/15. 10.1358/dnp.2010.23.9.1507297 21152452

[8] An Y, Varma VR, Varma S, Casanova R, Dammer E, Pletnikova O, et al (2018) Evidence for Brain Glucose Dysregulation in Alzheimer’s Disease. Alzheimer’s and Dementia. 14(3):318–329. 10.1016/j.jalz.2017.09.011 PMC5866736 29055815

[9] Varma VR, Oommen AM, Varma S, Casanova R, An Y, Andrews RM, et al Brain and blood metabolite signatures of pathology and progression in Alzheimer disease: A targeted metabolomics study. PLoS Med. 2018;15(1):e1002482 Epub 2018/01/26. 10.1371/journal.pmed.1002482 29370177 PMC5784884

[10] Snowden SG, Ebshiana AA, Hye A, An Y, Pletnikova O, O’Brien R, et al Association between fatty acid metabolism in the brain and Alzheimer disease neuropathology and cognitive performance: A nontargeted metabolomic study. PLoS Med. 2017;14(3):e1002266 Epub 2017/03/23. 10.1371/journal.pmed.1002266 28323825 PMC5360226

[11] Kihara T, Shimohama S. Alzheimer’s disease and acetylcholine receptors. Acta Neurobiol Exp (Wars). 2004;64(1):99–105. Epub 2004/06/12. 15190684 10.55782/ane-2004-1495

[12] Bazzari FH, Abdallah DM, El-Abhar HS. Pharmacological Interventions to Attenuate Alzheimer’s Disease Progression: The Story So Far. Curr Alzheimer Res. 2019. Epub 2019/03/05. 10.2174/1567205016666190301111120 30827243

[13] Fuso A, Scarpa S. One-carbon metabolism and Alzheimer’s disease: is it all a methylation matter? Neurobiol Aging. 2011;32(7):1192–5. Epub 2011/04/29. 10.1016/j.neurobiolaging.2011.01.012 21524430

[14] Whiley L, Sen A, Heaton J, Proitsi P, Garcia-Gomez D, Leung R, et al Evidence of altered phosphatidylcholine metabolism in Alzheimer’s disease. Neurobiol Aging. 2014;35(2):271–8. Epub 2013/09/18. 10.1016/j.neurobiolaging.2013.08.001 24041970 PMC5866043

[15] Madeo F, Eisenberg T, Pietrocola F, Kroemer G. Spermidine in health and disease. Science. 2018;359(6374). Epub 2018/01/27. 10.1126/science.aan2788 29371440

[16] Graham SF, Chevallier OP, Elliott CT, Holscher C, Johnston J, McGuinness B, et al Untargeted metabolomic analysis of human plasma indicates differentially affected polyamine and L-arginine metabolism in mild cognitive impairment subjects converting to Alzheimer’s disease. PLoS ONE. 2015;10(3):e0119452 Epub 2015/03/25. 10.1371/journal.pone.0119452 25803028 PMC4372431

[17] Roe AJ, Zhang S, Bhadelia RA, Johnson EJ, Lichtenstein AH, Rogers GT, et al Choline and its metabolites are differently associated with cardiometabolic risk factors, history of cardiovascular disease, and MRI-documented cerebrovascular disease in older adults. Am J Clin Nutr. 2017;105(6):1283–90. Epub 2017/03/31. 10.3945/ajcn.116.137158 28356272 PMC5445668

[18] Bekdash RA. Choline, the brain and neurodegeneration: insights from epigenetics. Front Biosci (Landmark Ed). 2018;23:1113–43. Epub 2017/09/21. 28930592 10.2741/4636

[19] Mandal PK, Saharan S, Tripathi M, Murari G. Brain glutathione levels—a novel biomarker for mild cognitive impairment and Alzheimer’s disease. Biol Psychiatry. 2015;78(10):702–10. Epub 2015/05/25. 10.1016/j.biopsych.2015.04.005 26003861

[20] Ferrucci L. The Baltimore Longitudinal Study of Aging (BLSA): a 50-year-long journey and plans for the future. J Gerontol A Biol Sci Med Sci. 2008;63(12):1416–9. Epub 2009/01/08. 10.1093/gerona/63.12.1416 19126858 PMC5004590

[21] O’Brien RJ, Resnick SM, Zonderman AB, Ferrucci L, Crain BJ, Pletnikova O, et al Neuropathologic studies of the Baltimore Longitudinal Study of Aging (BLSA). J Alzheimers Dis. 2009;18(3):665–75. Epub 2009/08/08. 10.3233/JAD-2009-1179 19661626 PMC2978421

[22] Gamaldo A, Moghekar A, Kilada S, Resnick SM, Zonderman AB, O’Brien R. Effect of a clinical stroke on the risk of dementia in a prospective cohort. Neurology. 2006;67(8):1363–9. Epub 2006/10/25. 10.1212/01.wnl.0000240285.89067.3f 17060561

[23] Troncoso JC, Zonderman AB, Resnick SM, Crain B, Pletnikova O, O’Brien RJ. Effect of infarcts on dementia in the Baltimore longitudinal study of aging. Ann Neurol. 2008;64(2):168–76. Epub 2008/05/23. 10.1002/ana.21413 18496870 PMC2694129

[24] Mirra SS, Heyman A, McKeel D, Sumi SM, Crain BJ, Brownlee LM, et al The Consortium to Establish a Registry for Alzheimer’s Disease (CERAD). Part II. Standardization of the neuropathologic assessment of Alzheimer’s disease. Neurology. 1991;41(4):479–86. Epub 1991/04/01. 10.1212/wnl.41.4.479 2011243

[25] Braak H, Braak E. Neuropathological stageing of Alzheimer-related changes. Acta Neuropathol. 1991;82(4):239–59. Epub 1991/01/01. 10.1007/bf00308809 1759558

[26] Iacono D, Resnick SM, O’Brien R, Zonderman AB, An Y, Pletnikova O, et al Mild cognitive impairment and asymptomatic Alzheimer disease subjects: equivalent beta-amyloid and tau loads with divergent cognitive outcomes. J Neuropathol Exp Neurol. 2014;73(4):295–304. Epub 2014/03/13. 10.1097/NEN.0000000000000052 24607960 PMC4062187

[27] Reitz C, Honig L, Vonsattel JP, Tang MX, Mayeux R. Memory performance is related to amyloid and tau pathology in the hippocampus. J Neurol Neurosurg Psychiatry. 2009;80(7):715–21. Epub 2009/03/05. 10.1136/jnnp.2008.154146 19258354 PMC2785022

[28] Knopman DS, Lundt ES, Therneau TM, Vemuri P, Lowe VJ, Kantarci K, et al Entorhinal cortex tau, amyloid-beta, cortical thickness and memory performance in non-demented subjects. Brain. 2019. Epub 2019/02/14. 10.1093/brain/awz025 PMC6439321 30759182

[29] Hyman BT, Van Hoesen GW, Damasio AR, Barnes CL. Alzheimer’s disease: cell-specific pathology isolates the hippocampal formation. Science. 1984;225(4667):1168–70. Epub 1984/09/14. 10.1126/science.6474172 6474172

[30] Buckley RF, Hanseeuw B, Schultz AP, Vannini P, Aghjayan SL, Properzi MJ, et al Region-Specific Association of Subjective Cognitive Decline With Tauopathy Independent of Global beta-Amyloid Burden. JAMA Neurol. 2017;74(12):1455–63. Epub 2017/10/04. 10.1001/jamaneurol.2017.2216 28973551 PMC5774633

[31] Li Y, Rinne JO, Mosconi L, Pirraglia E, Rusinek H, DeSanti S, et al Regional analysis of FDG and PIB-PET images in normal aging, mild cognitive impairment, and Alzheimer’s disease. Eur J Nucl Med Mol Imaging. 2008;35(12):2169–81. Epub 2008/06/21. 10.1007/s00259-008-0833-y 18566819 PMC2693402

[32] Larner AJ. The cerebellum in Alzheimer’s disease. Dement Geriatr Cogn Disord. 1997;8(4):203–9. Epub 1997/07/01. 10.1159/000106632 9213064

[33] Koike S, Bundo M, Iwamoto K, Suga M, Kuwabara H, Ohashi Y, et al A snapshot of plasma metabolites in first-episode schizophrenia: a capillary electrophoresis time-of-flight mass spectrometry study. Transl Psychiatry. 2014;4:e379 Epub 2014/04/10. 10.1038/tp.2014.19 24713860 PMC4012283

[34] Fujii T, Hattori K, Miyakawa T, Ohashi Y, Sato H, Kunugi H. Metabolic profile alterations in the postmortem brains of patients with schizophrenia using capillary electrophoresis-mass spectrometry. Schizophr Res. 2017;183:70–4. Epub 2016/11/20. 10.1016/j.schres.2016.11.011 27856156

[35] Soga T, Heiger DN. Amino acid analysis by capillary electrophoresis electrospray ionization mass spectrometry. Anal Chem. 2000;72(6):1236–41. Epub 2000/03/31. 10.1021/ac990976y 10740865

[36] Soga T, Ueno Y, Naraoka H, Ohashi Y, Tomita M, Nishioka T. Simultaneous determination of anionic intermediates for Bacillus subtilis metabolic pathways by capillary electrophoresis electrospray ionization mass spectrometry. Anal Chem. 2002;74(10):2233–9. Epub 2002/06/01. 10.1021/ac020064n 12038746

[37] Soga T, Ohashi Y, Ueno Y, Naraoka H, Tomita M, Nishioka T. Quantitative metabolome analysis using capillary electrophoresis mass spectrometry. J Proteome Res. 2003;2(5):488–94. Epub 2003/10/30. 10.1021/pr034020m 14582645

[38] Ohashi Y, Hirayama A, Ishikawa T, Nakamura S, Shimizu K, Ueno Y, et al Depiction of metabolome changes in histidine-starved Escherichia coli by CE-TOFMS. Mol Biosyst. 2008;4(2):135–47. Epub 2008/01/24. 10.1039/b714176a 18213407

[39] Sasaki K, Sagawa H, Suzuki M, Yamamoto H, Tomita M, Soga T, et al Metabolomics Platform with Capillary Electrophoresis Coupled with High-Resolution Mass Spectrometry for Plasma Analysis. Anal Chem. 2019;91(2):1295–301. Epub 2018/12/01. 10.1021/acs.analchem.8b02994 30500154

[40] Soga T, Igarashi K, Ito C, Mizobuchi K, Zimmermann HP, Tomita M. Metabolomic profiling of anionic metabolites by capillary electrophoresis mass spectrometry. Anal Chem. 2009;81(15):6165–74. Epub 2009/06/16. 10.1021/ac900675k 19522513

[41] Benjamini Y, Hochberg Y. Controlling the False Discovery Rate: A Practical and Powerful Approach to Multiple Testing. Journal of the Royal Statistical Society Series B (Methodological). 1995;57(1):289–300.

[42] Lee J. Statistical bioinformatics. 1et ed New Jersey: John Wiley & Sons Inc.; 2010.

[43] Irizarry RA, Hobbs B, Collin F, Beazer-Barclay YD, Antonellis KJ, Scherf U, et al Exploration, normalization, and summaries of high density oligonucleotide array probe level data. Biostatistics. 2003;4(2):249–64. Epub 2003/08/20. 10.1093/biostatistics/4.2.249 12925520

[44] Dai M, Wang P, Boyd AD, Kostov G, Athey B, Jones EG, et al Evolving gene/transcript definitions significantly alter the interpretation of GeneChip data. Nucleic Acids Res. 2005;33(20):e175 Epub 2005/11/15. 10.1093/nar/gni179 16284200 PMC1283542

[45] Ritchie ME, Phipson B, Wu D, Hu Y, Law CW, Shi W, et al limma powers differential expression analyses for RNA-sequencing and microarray studies. Nucleic Acids Res. 2015;43(7):e47 Epub 2015/01/22. 10.1093/nar/gkv007 25605792 PMC4402510

[46] Paglia G, Stocchero M, Cacciatore S, Lai S, Angel P, Alam MT, et al Unbiased Metabolomic Investigation of Alzheimer’s Disease Brain Points to Dysregulation of Mitochondrial Aspartate Metabolism. J Proteome Res. 2016;15(2):608–18. Epub 2015/12/31. 10.1021/acs.jproteome.5b01020 26717242 PMC5751881

[47] Wood PL, Etienne P, Lal S, Nair NP, Finlayson MH, Gauthier S, et al A post-mortem comparison of the cortical cholinergic system in Alzheimer’s disease and Pick’s disease. J Neurol Sci. 1983;62(1–3):211–7. Epub 1983/12/01. 10.1016/0022-510x(83)90200-9 6142096

[48] Yates CM, Simpson J, Gordon A. Regional brain 5-hydroxytryptamine levels are reduced in senile Down’s syndrome as in Alzheimer’s disease. Neurosci Lett. 1986;65(2):189–92. Epub 1986/04/11. 10.1016/0304-3940(86)90302-2 2940479

[49] Foster NL, Tamminga CA, O’Donohue TL, Tanimoto K, Bird ED, Chase TN. Brain choline acetyltransferase activity and neuropeptide Y concentrations in Alzheimer’s disease. Neurosci Lett. 1986;63(1):71–5. Epub 1986/01/02. 10.1016/0304-3940(86)90015-7 3754039

[50] Rossor MN, Garrett NJ, Johnson AL, Mountjoy CQ, Roth M, Iversen LL. A post-mortem study of the cholinergic and GABA systems in senile dementia. Brain. 1982;105(Pt 2):313–30. Epub 1982/06/01. 10.1093/brain/105.2.313 7082992

[51] Wang H, Tan L, Wang HF, Liu Y, Yin RH, Wang WY, et al Magnetic Resonance Spectroscopy in Alzheimer’s Disease: Systematic Review and Meta-Analysis. J Alzheimers Dis. 2015;46(4):1049–70. Epub 2015/09/25. 10.3233/JAD-143225 26402632

[52] Ganz AB, Cohen VV, Swersky CC, Stover J, Vitiello GA, Lovesky J, et al Genetic Variation in Choline-Metabolizing Enzymes Alters Choline Metabolism in Young Women Consuming Choline Intakes Meeting Current Recommendations. Int J Mol Sci. 2017;18(2). Epub 2017/01/31. 10.3390/ijms18020252 PMC5343788 28134761

[53] Chiang PK, Gordon RK, Tal J, Zeng GC, Doctor BP, Pardhasaradhi K, et al S-Adenosylmethionine and methylation. FASEB J. 1996;10(4):471–80. Epub 1996/03/01. 8647346

[54] Field MS, Shields KS, Abarinov EV, Malysheva OV, Allen RH, Stabler SP, et al Reduced MTHFD1 activity in male mice perturbs folate- and choline-dependent one-carbon metabolism as well as transsulfuration. J Nutr. 2013;143(1):41–5. Epub 2012/11/30. 10.3945/jn.112.169821 23190757 PMC3521460

[55] Ma S, Gladyshev VN. Molecular signatures of longevity: Insights from cross-species comparative studies. Semin Cell Dev Biol. 2017;70:190–203. Epub 2017/08/13. 10.1016/j.semcdb.2017.08.007 28800931 PMC5807068

[56] Lee BC, Kaya A, Gladyshev VN. Methionine restriction and life-span control. Ann N Y Acad Sci. 2016;1363:116–24. Epub 2015/12/15. 10.1111/nyas.12973 26663138 PMC5008916

[57] Tapia-Rojas C, Lindsay CB, Montecinos-Oliva C, Arrazola MS, Retamales RM, Bunout D, et al Is L-methionine a trigger factor for Alzheimer’s-like neurodegeneration?: Changes in Abeta oligomers, tau phosphorylation, synaptic proteins, Wnt signaling and behavioral impairment in wild-type mice. Mol Neurodegener. 2015;10:62 Epub 2015/11/23. 10.1186/s13024-015-0057-0 26590557 PMC4654847

[58] Lopez LM, Harris SE, Luciano M, Liewald D, Davies G, Gow AJ, et al Evolutionary conserved longevity genes and human cognitive abilities in elderly cohorts. Eur J Hum Genet. 2012;20(3):341–7. Epub 2011/11/03. 10.1038/ejhg.2011.201 22045296 PMC3283186

[59] Hainsworth AH, Yeo NE, Weekman EM, Wilcock DM. Homocysteine, hyperhomocysteinemia and vascular contributions to cognitive impairment and dementia (VCID). Biochim Biophys Acta. 2016;1862(5):1008–17. Epub 2015/12/23. 10.1016/j.bbadis.2015.11.015 26689889 PMC4821788

[60] Chon J, Stover PJ, Field MS. Targeting nuclear thymidylate biosynthesis. Mol Aspects Med. 2017;53:48–56. Epub 2016/11/24. 10.1016/j.mam.2016.11.005 27876557 PMC5253096

[61] Hou Y, Song H, Croteau DL, Akbari M, Bohr VA. Genome instability in Alzheimer disease. Mech Ageing Dev. 2017;161(Pt A):83–94. Epub 2016/04/24. 10.1016/j.mad.2016.04.005 27105872 PMC5195918

[62] Garcia-Gimenez JL, Roma-Mateo C, Perez-Machado G, Peiro-Chova L, Pallardo FV. Role of glutathione in the regulation of epigenetic mechanisms in disease. Free Radic Biol Med. 2017;112:36–48. Epub 2017/07/15. 10.1016/j.freeradbiomed.2017.07.008 28705657

[63] Aoyama K, Nakaki T. Impaired glutathione synthesis in neurodegeneration. Int J Mol Sci. 2013;14(10):21021–44. Epub 2013/10/23. 10.3390/ijms141021021 24145751 PMC3821656

[64] Duffy SL, Lagopoulos J, Hickie IB, Diamond K, Graeber MB, Lewis SJ, et al Glutathione relates to neuropsychological functioning in mild cognitive impairment. Alzheimers Dement. 2014;10(1):67–75. Epub 2013/05/22. 23688577 10.1016/j.jalz.2013.01.005

[65] Pan X, Nasaruddin MB, Elliott CT, McGuinness B, Passmore AP, Kehoe PG, et al Alzheimer’s disease-like pathology has transient effects on the brain and blood metabolome. Neurobiol Aging. 2016;38:151–63. Epub 2016/02/02. 10.1016/j.neurobiolaging.2015.11.014 26827653

[66] Gamble LD, Hogarty MD, Liu X, Ziegler DS, Marshall G, Norris MD, et al Polyamine pathway inhibition as a novel therapeutic approach to treating neuroblastoma. Front Oncol. 2012;2:162 Epub 2012/11/28. 10.3389/fonc.2012.00162 23181218 PMC3499881

[67] Danysz W, Parsons CG. Alzheimer’s disease, beta-amyloid, glutamate, NMDA receptors and memantine—searching for the connections. Br J Pharmacol. 2012;167(2):324–52. Epub 2012/06/01. 10.1111/j.1476-5381.2012.02057.x 22646481 PMC3481041

[68] Gilad GM, Gilad VH. Overview of the brain polyamine-stress-response: regulation, development, and modulation by lithium and role in cell survival. Cell Mol Neurobiol. 2003;23(4–5):637–49. Epub 2003/09/30. 10.1023/a:1025036532672 14514021 PMC11530194

[69] Pegg AE. Functions of Polyamines in Mammals. J Biol Chem. 2016;291(29):14904–12. Epub 2016/06/09. 10.1074/jbc.R116.731661 27268251 PMC4946908

[70] Skatchkov SN, Woodbury-Farina MA, Eaton M. The role of glia in stress: polyamines and brain disorders. Psychiatr Clin North Am. 2014;37(4):653–78. Epub 2014/12/03. 10.1016/j.psc.2014.08.008 25455070 PMC4394863

[71] Pantazatos SP, Andrews SJ, Dunning-Broadbent J, Pang J, Huang YY, Arango V, et al Isoform-level brain expression profiling of the spermidine/spermine N1-Acetyltransferase1 (SAT1) gene in major depression and suicide. Neurobiol Dis. 2015;79:123–34. Epub 2015/05/12. 10.1016/j.nbd.2015.04.014 25959060 PMC4834874

[72] Limon A, Mamdani F, Hjelm BE, Vawter MP, Sequeira A. Targets of polyamine dysregulation in major depression and suicide: Activity-dependent feedback, excitability, and neurotransmission. Neurosci Biobehav Rev. 2016;66:80–91. Epub 2016/04/26. 10.1016/j.neubiorev.2016.04.010 27108532 PMC5096383

[73] Zahedi K, Huttinger F, Morrison R, Murray-Stewart T, Casero RA, Strauss KI. Polyamine catabolism is enhanced after traumatic brain injury. J Neurotrauma. 2010;27(3):515–25. Epub 2009/12/09. 10.1089/neu.2009.1097 19968558 PMC2867553

[74] Wood PL, Khan MA, Kulow SR, Mahmood SA, Moskal JR. Neurotoxicity of reactive aldehydes: the concept of "aldehyde load" as demonstrated by neuroprotection with hydroxylamines. Brain Res. 2006;1095(1):190–9. Epub 2006/05/30. 10.1016/j.brainres.2006.04.038 16730673

[75] Fan J, Chen M, Wang X, Tian Z, Wang J, Fan D, et al Targeting Smox is neuroprotective and ameliorates brain inflammation in cerebral ischemia/reperfusion rats. Toxicol Sci. 2018. Epub 2018/12/24. 10.1093/toxsci/kfy300 30576531

[76] Hansmannel F, Sillaire A, Kamboh MI, Lendon C, Pasquier F, Hannequin D, et al Is the urea cycle involved in Alzheimer’s disease? J Alzheimers Dis. 2010;21(3):1013–21. Epub 2010/08/10. 10.3233/JAD-2010-100630 20693631 PMC2945690

[77] Seiler N, Daune-Anglard G. Endogenous ornithine in search for CNS functions and therapeutic applications. Metab Brain Dis. 1993;8(3):151–79. Epub 1993/09/01. 10.1007/bf00996928 8272027

[78] Jesko H, Lukiw WJ, Wilkaniec A, Cieslik M, Gassowska-Dobrowolska M, Murawska E, et al Altered Expression of Urea Cycle Enzymes in Amyloid-beta Protein Precursor Overexpressing PC12 Cells and in Sporadic Alzheimer’s Disease Brain. J Alzheimers Dis. 2018;62(1):279–91. Epub 2018/02/15. 10.3233/JAD-170427 29439324

[79] Moffett JR, Ross B, Arun P, Madhavarao CN, Namboodiri AM. N-Acetylaspartate in the CNS: from neurodiagnostics to neurobiology. Prog Neurobiol. 2007;81(2):89–131. Epub 2007/02/06. 10.1016/j.pneurobio.2006.12.003 17275978 PMC1919520

[80] Clark JB. N-acetyl aspartate: a marker for neuronal loss or mitochondrial dysfunction. Dev Neurosci. 1998;20(4–5):271–6. Epub 1998/10/21. 10.1159/000017321 9778562

[81] Zaroff S, Leone P, Markov V, Francis JS. Transcriptional regulation of N-acetylaspartate metabolism in the 5xFAD model of Alzheimer’s disease: evidence for neuron-glia communication during energetic crisis. Mol Cell Neurosci. 2015;65:143–52. Epub 2015/03/15. 10.1016/j.mcn.2015.03.009 25766789 PMC4393791

[82] Yudkoff M, Nelson D, Daikhin Y, Erecinska M. Tricarboxylic acid cycle in rat brain synaptosomes. Fluxes and interactions with aspartate aminotransferase and malate/aspartate shuttle. J Biol Chem. 1994;269(44):27414–20. Epub 1994/11/04. 7961653

[83] Arai H, Kobayashi K, Ichimiya Y, Kosaka K, Iizuka R. A preliminary study of free amino acids in the postmortem temporal cortex from Alzheimer-type dementia patients. Neurobiol Aging. 1984;5(4):319–21. Epub 1984/01/01. 10.1016/0197-4580(84)90009-5 6152305

[84] Ambrad Giovannetti E, Fuhrmann M. Unsupervised excitation: GABAergic dysfunctions in Alzheimer’s disease. Brain Res. 2019;1707:216–26. Epub 2018/12/07. 10.1016/j.brainres.2018.11.042 30503351

[85] Gueli MC, Taibi G. Alzheimer’s disease: amino acid levels and brain metabolic status. Neurol Sci. 2013;34(9):1575–9. Epub 2013/01/29. 10.1007/s10072-013-1289-9 23354600

[86] Mucke L, Selkoe DJ. Neurotoxicity of amyloid beta-protein: synaptic and network dysfunction. Cold Spring Harb Perspect Med. 2012;2(7):a006338 Epub 2012/07/05. 10.1101/cshperspect.a006338 22762015 PMC3385944

[87] Saura J, Luque JM, Cesura AM, Da Prada M, Chan-Palay V, Huber G, et al Increased monoamine oxidase B activity in plaque-associated astrocytes of Alzheimer brains revealed by quantitative enzyme radioautography. Neuroscience. 1994;62(1):15–30. Epub 1994/09/01. 10.1016/0306-4522(94)90311-5 7816197

[88] Carter SF, Herholz K, Rosa-Neto P, Pellerin L, Nordberg A, Zimmer ER. Astrocyte Biomarkers in Alzheimer’s Disease. Trends Mol Med. 2019;25(2):77–95. Epub 2019/01/07. 10.1016/j.molmed.2018.11.006 30611668

[89] Schedin-Weiss S, Inoue M, Hromadkova L, Teranishi Y, Yamamoto NG, Wiehager B, et al Monoamine oxidase B is elevated in Alzheimer disease neurons, is associated with gamma-secretase and regulates neuronal amyloid beta-peptide levels. Alzheimers Res Ther. 2017;9(1):57 Epub 2017/08/03. 10.1186/s13195-017-0279-1 28764767 PMC5540560

[90] Jo S, Yarishkin O, Hwang YJ, Chun YE, Park M, Woo DH, et al GABA from reactive astrocytes impairs memory in mouse models of Alzheimer’s disease. Nat Med. 2014;20(8):886–96. Epub 2014/06/30. 10.1038/nm.3639 24973918 PMC8385452

[91] Heja L, Nyitrai G, Kekesi O, Dobolyi A, Szabo P, Fiath R, et al Astrocytes convert network excitation to tonic inhibition of neurons. BMC Biol. 2012;10:26 Epub 2012/03/17. 10.1186/1741-7007-10-26 22420899 PMC3342137

[92] Yoon BE, Woo J, Chun YE, Chun H, Jo S, Bae JY, et al Glial GABA, synthesized by monoamine oxidase B, mediates tonic inhibition. J Physiol. 2014;592(22):4951–68. Epub 2014/09/23. 10.1113/jphysiol.2014.278754 25239459 PMC4259537

[93] Ivanova S, Batliwalla F, Mocco J, Kiss S, Huang J, Mack W, et al Neuroprotection in cerebral ischemia by neutralization of 3-aminopropanal. Proc Natl Acad Sci U S A. 2002;99(8):5579–84. Epub 2002/04/12. 10.1073/pnas.082609299 11943872 PMC122812

[94] Yu Z, Li W, Hillman J, Brunk UT. Human neuroblastoma (SH-SY5Y) cells are highly sensitive to the lysosomotropic aldehyde 3-aminopropanal. Brain Res. 2004;1016(2):163–9. Epub 2004/07/13. 10.1016/j.brainres.2004.04.075 15246852

[95] Wood PL, Khan MA, Moskal JR. The concept of "aldehyde load" in neurodegenerative mechanisms: cytotoxicity of the polyamine degradation products hydrogen peroxide, acrolein, 3-aminopropanal, 3-acetamidopropanal and 4-aminobutanal in a retinal ganglion cell line. Brain Res. 2007;1145:150–6. Epub 2007/03/17. 10.1016/j.brainres.2006.10.004 17362887

[96] Faber J, Fonseca LM. How sample size influences research outcomes. Dental Press J Orthod. 2014;19(4):27–9. Epub 2014/10/04. 10.1590/2176-9451.19.4.027-029.ebo 25279518 PMC4296634

[97] Button KS, Ioannidis JP, Mokrysz C, Nosek BA, Flint J, Robinson ES, et al Power failure: why small sample size undermines the reliability of neuroscience. Nat Rev Neurosci. 2013;14(5):365–76. Epub 2013/04/11. 10.1038/nrn3475 23571845

