# Correction: Dysregulation of multiple metabolic networks related to brain transmethylation and polyamine pathways in Alzheimer disease: A targeted metabolomic and transcriptomic study

**DOI:** 10.1371/journal.pmed.1003439

**Published:** 2020-10-21

**Authors:** Uma V. Mahajan, Vijay R. Varma, Michael E. Griswold, Chad T. Blackshear, Yang An, Anup M. Oommen, Sudhir Varma, Juan C. Troncoso, Olga Pletnikova, Richard O’Brien, Timothy J. Hohman, Cristina Legido-Quigley, Madhav Thambisetty

During the process of revising the figures and ensuring they met the required standards for resolution/size, some of the line alignments in Fig 11 were lost or misaligned. See the corrected Fig 11 here.

**Fig 11:**
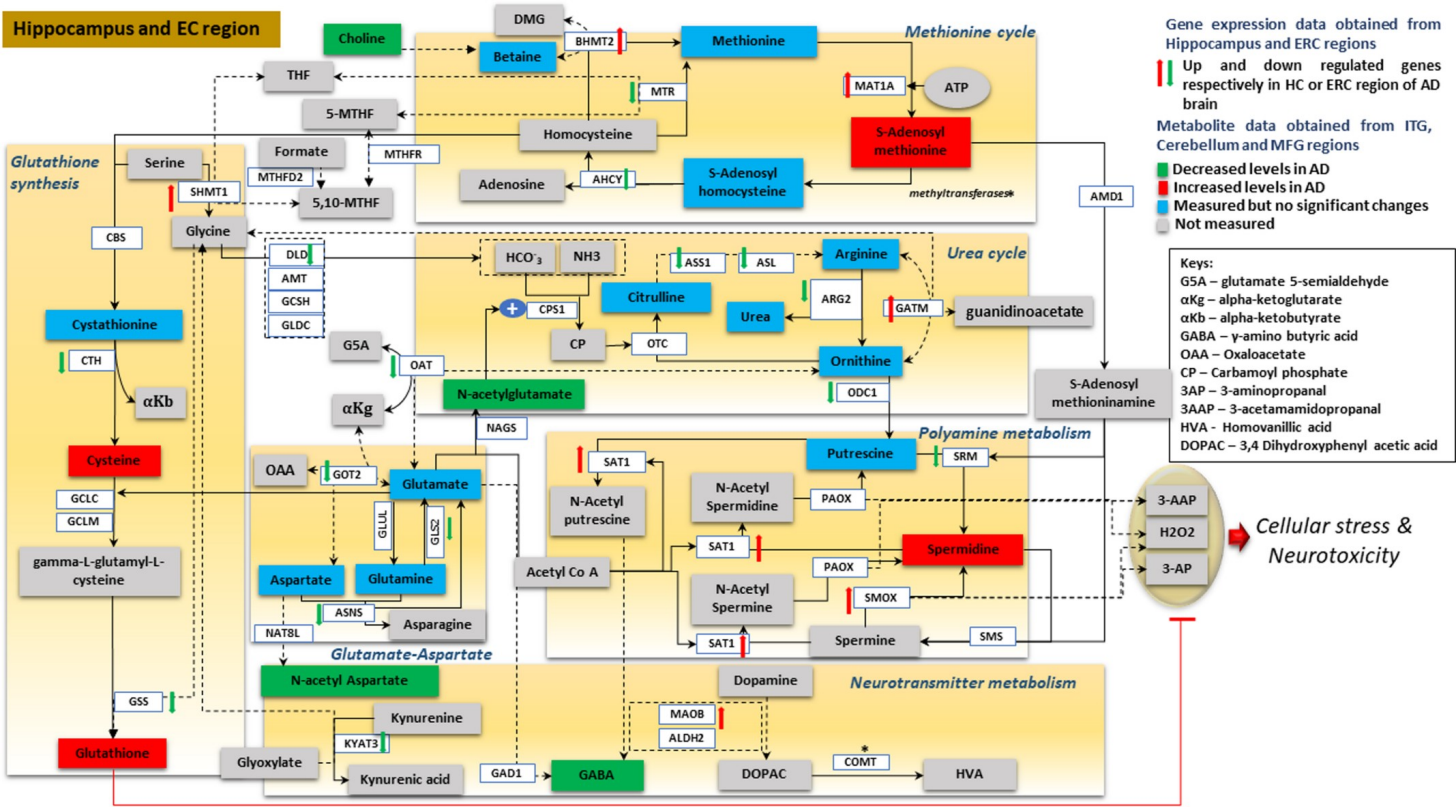
Integrated summary of alterations in metabolite concentrations and gene expression within metabolic pathways linked to transmethylation and polyamine synthesis/catabolism. Reactions within each of the six categories related to transmethylation and polyamine metabolism are shown within brown boxes [93–95]. Metabolites whose concentrations are increased in the AD ITG relative to controls are shown in red boxes; those that are reduced in AD are shown in green boxes. Metabolites whose concentrations were measured but did not differ between AD and control are indicated in blue boxes; metabolites whose concentrations were not assayed are shown in gray boxes. Red up and green down arrows indicate significantly increased and reduced gene expression in the ERC/hippocampus, respectively. AD, Alzheimer disease; AHCY, adenosylhomocysteinase; ALDH2, aldehyde dehydrogenase 2; AMD1, adenosylmethionine decarboxylase 1; AMT, aminomethyltransferase; ARG2, arginase 2; ASL, argininosuccinate lyase; ASNS, asparagine synthetase; ASS1, argininosuccinate synthase 1; BHMT2, betaine homocysteine smethyltransferase 2; CBS, cystathionine beta synthase; CN, Control; COMT, catechol-o-methyltransferase; CPS1, carbamoyl phosphate synthase 1; CTH, cystathionine gamma-lyase; DLD, dihydrolipoamide dehydrogenase; ERC, entorhinal cortex; GAD1, glutamate decarboxylase 1; GATM, glycine amidino transferase; GCLC, glutamate cysteine ligase catalytic; GCLM, glutamate cysteine ligase modifier; GCSH, glycine cleavage system protein H; GLDC, glycine decarboxylase; GLS2, glutaminase 2; GLUL, glutamine synthetase; GOT2, glutamic oxaloacetic transaminase 2; GSS, GSH synthetase; HC, hippocampus; ITG, inferior temporal gyrus; KYAT3, kynurenine aminotransferase 3; MAT1A, methionine adenosyltransferase 1A; MFG, middle frontal gyrus; MOAB, monoamine oxidase B; MTHFD2, methylenetetrahydrofolate dehydrogenase 2; MTHFR, methylenetetrahydrofolate reductase; MTR, methyltransferase; NAT8L, n-acetyltransferase 8 like; OAT, ornithine aminotransferase; ODC1, ornithine decarboxylase 1; OTC, ornithine carbamoyltransferase; PAOX, polyamine oxidase; SAT1, spermidine/spermine n1-acetyltransferase 1; SHMT1, serine hydroxymethyl transferase 1; SMOX, spermine oxidase; SMS, spermine synthase; SRM, spermidine synthase.
