# Supplementary material for: Dysregulation of multiple metabolic networks related to brain transmethylation and polyamine pathways in Alzheimer disease: A targeted metabolomic and transcriptomic study
Source: PLoS Med. 2020 Jan 24;17(1):e1003012. doi: 10.1371/journal.pmed.1003012 (PMC6980402; doi:10.1371/journal.pmed.1003012)
Supplement: S1 STROBE Checklist — (DOCX) [file pmed.1003012.s001.docx]

**S1 STROBE (Strengthening the Reporting of Observational Studies in Epidemiology) Guideline Checklist**

STROBE Statement—checklist of items that should be included in reports of observational studies

|  | Item No. | Recommendation | Paragraph number in section | | Relevant text from manuscript | |
| --- | --- | --- | --- | --- | --- | --- |
| **Title and abstract** | 1 | (*a*) Indicate the study’s design with a commonly used term in the title or the abstract | Title | | a targeted metabolomic and transcriptomic study | |
|  |  | (*b*) Provide in the abstract an informative and balanced summary of what was done and what was found | Paragraph 1-3 in Abstract | | See Section: Abstract | |
| Introduction | | | |  | |  |
| Background/rationale | 2 | Explain the scientific background and rationale for the investigation being reported | Paragraph 1 in introduction | | A growing body of evidence suggests that Alzheimer’s disease (AD) is a pervasive metabolic disorder with dysregulation of multiple biochemical pathways that may be associated with both severity of AD pathology… | |
| Objectives | 3 | State specific objectives, including any prespecified hypotheses | Paragraph 3 in Introduction | | In this study, we therefore tested the hypothesis that dysregulation of choline-related biochemical pathways in the brain are associated with AD pathogenesis… | |
| Methods | | | |  | |  |
| Study design | 4 | Present key elements of study design early in the paper | First 4 subsections in Methods | | See section: Methods:  - Participants  - Brain Tissue  - Metabolomic profiling  - Gene expression | |
| Setting | 5 | Describe the setting, locations, and relevant dates, including periods of recruitment, exposure, follow-up, and data collection | Paragraph 1 in Participants subsection in Methods | | The National Institute on Aging’s (NIA) Baltimore Longitudinal Study of Aging (BLSA), is one of the longest running scientific studies of human aging in the U.S… | |
| Participants | 6 | (*a*) *Cohort study*—Give the eligibility criteria, and the sources and methods of selection of participants. Describe methods of follow-up  *Case-control study*—Give the eligibility criteria, and the sources and methods of case ascertainment and control selection. Give the rationale for the choice of cases and controls  *Cross-sectional study*—Give the eligibility criteria, and the sources and methods of selection of participants | Paragraphs 1-2 in Participants subsection in Methods | | This observational study began in 1958 and includes longitudinal clinical, radiological and laboratory evaluations on community dwelling volunteer participants. Participants are assessed…  Autopsy participants were classified within three groups based on the following criteria... | |
|  |  | (*b*) *Cohort study*—For matched studies, give matching criteria and number of exposed and unexposed  *Case-control study*—For matched studies, give matching criteria and the number of controls per case | Paragraph 2 in Participants subsection in Methods | | AD participants (n=17) had a clinical diagnosis of either AD or MCI due to AD within 1 year of death and a CERAD pathology score of >1 (i.e. CERAD B or C);… | |
| Variables | 7 | Clearly define all outcomes, exposures, predictors, potential confounders, and effect modifiers. Give diagnostic criteria, if applicable | First 4 subsections in Methods | | See section: Methods:  - Participants  - Brain Tissue  - Metabolomic profiling  - Gene expression | |
| Data sources/ measurement | 8* | For each variable of interest, give sources of data and details of methods of assessment (measurement). Describe comparability of assessment methods if there is more than one group | First 4 subsections in Methods | | See section: Methods:  - Participants  - Brain Tissue  - Metabolomic profiling  - Gene expression | |
| Bias | 9 | Describe any efforts to address potential sources of bias | N/A | |  | |
| Study size | 10 | Explain how the study size was arrived at | N/A | |  | |

| Quantitative variables | 11 | Explain how quantitative variables were handled in the analyses. If applicable, describe which groupings were chosen and why | Fifth subsection, Statistical Analyses, under Methods | See section: Statistical Analysis | |  |
| --- | --- | --- | --- | --- | --- | --- |
| Statistical methods | 12 | (*a*) Describe all statistical methods, including those used to control for confounding | Fifth subsection, Statistical Analyses, under Methods | See section: Statistical Analysis | |  |
|  |  | (*b*) Describe any methods used to examine subgroups and interactions | N/A |  | |  |
|  |  | (*c*) Explain how missing data were addressed | Third paragraph under fifth subsection, Statistical Analyses, under Methods | As in our previous metabolomics analyses (9), metabolites with greater than 30% of values missing were dropped from all analyses. From the a priori defined 27 principal metabolites in the transmethylation and polyamine pathways, 1 metabolite (spermine) was dropped due to missingness >30%. For the remaining metabolites (i.e. 26 in total), values indicated as less than the limit of detection were imputed as the lowest detectable value divided by 2 (1.15% of metabolites imputed on average). | |  |
|  |  | (*d*) *Cohort study*—If applicable, explain how loss to follow-up was addressed  *Case-control study*—If applicable, explain how matching of cases and controls was addressed  *Cross-sectional study*—If applicable, describe analytical methods taking account of sampling strategy | N/A |  | |  |
|  |  | (*e*) Describe any sensitivity analyses | Last sentence in fourth paragraph under fifth subsection, Statistical Analyses, under Methods | Sensitivity analyses using generalized estimating equation (GEE) clustered analyses and non-parametric Spearman correlations with independent Kruskal-Wallis tests gave similar results. |  |  |
| Results | | | | | | |
| Participants | 13* | (a) Report numbers of individuals at each stage of study—eg numbers potentially eligible, examined for eligibility, confirmed eligible, included in the study, completing follow-up, and analysed | NA |  | |  |
|  |  | (b) Give reasons for non-participation at each stage | NA |  | |  |
|  |  | (c) Consider use of a flow diagram | NA |  | |  |
| Descriptive data | 14* | (a) Give characteristics of study participants (eg demographic, clinical, social) and information on exposures and potential confounders | 16 | The demographic characteristics of BLSA participants in the autopsy cohort…  Table 1A and 1B | |  |
|  |  | (b) Indicate number of participants with missing data for each variable of interest | NA |  | |  |
|  |  | (c) *Cohort study*—Summarise follow-up time (eg, average and total amount) | NA |  | |  |
| Outcome data | 15* | *Cohort study*—Report numbers of outcome events or summary measures over time | NA |  | |  |
|  |  | *Case-control study—*Report numbers in each exposure category, or summary measures of exposure | NA |  | |  |
|  |  | *Cross-sectional study—*Report numbers of outcome events or summary measures | Tables 2-4 in Results | Tables 2-4 | |  |
| Main results | 16 | (*a*) Give unadjusted estimates and, if applicable, confounder-adjusted estimates and their precision (eg, 95% confidence interval). Make clear which confounders were adjusted for and why they were included | Tables 2-4 in Results  Fifth subsection, Statistical Analyses, under Methods | Tables 2-4  See section Methods: Statistical Analysis | |  |
|  |  | (*b*) Report category boundaries when continuous variables were categorized | NA |  | |  |
|  |  | (*c*) If relevant, consider translating estimates of relative risk into absolute risk for a meaningful time period | NA |  | |  |

| Other analyses | 17 | Report other analyses done—eg analyses of subgroups and interactions, and sensitivity analyses | Tables S2-4 in Results  Fifth subsection, Statistical Analyses, under Methods | S2-S4 Tables &  See section Methods: Statistical Analysis |
| --- | --- | --- | --- | --- |
| Discussion | | | | |
| Key results | 18 | Summarise key results with reference to study objectives | First paragraph under Discussion | In this study, we substantially extended our prior work .... We examined whether dysregulation in the transmethylation pathway… |
| Limitations | 19 | Discuss limitations of the study, taking into account sources of potential bias or imprecision. Discuss both direction and magnitude of any potential bias | First paragraph under Conclusions and limitations subsection under Discussion | Some limitations of our study merit consideration. Our CE-MS based metabolomics assays… |
| Interpretation | 20 | Give a cautious overall interpretation of results considering objectives, limitations, multiplicity of analyses, results from similar studies, and other relevant evidence | Second paragraph under Conclusions and limitations subsection under Discussion | Our results implicate alterations in cellular methylation potential and increased flux in the transmethylation pathways, increased demand on antioxidant… |
| Generalisability | 21 | Discuss the generalisability (external validity) of the study results | First paragraph under Conclusions and limitations subsection under Discussion | Our interpretation of the results is therefore limited to the analytes that could be reliably detected |
| Other information | |  | | |
| Funding | 22 | Give the source of funding and the role of the funders for the present study and, if applicable, for the original study on which the present article is based | Under Acknoledgements | This research was supported in part by the Intramural Research Program of the NIH, National Institute on Aging… |

*Give information separately for cases and controls in case-control studies and, if applicable, for exposed and unexposed groups in cohort and cross-sectional studies.

**Note:** An Explanation and Elaboration article discusses each checklist item and gives methodological background and published examples of transparent reporting. The STROBE checklist is best used in conjunction with this article (freely available on the Web sites of PLoS Medicine at http://www.plosmedicine.org/, Annals of Internal Medicine at http://www.annals.org/, and Epidemiology at http://www.epidem.com/). Information on the STROBE Initiative is available at www.strobe-statement.org.
