# Supplementary material for: Dysregulation of multiple metabolic networks related to brain transmethylation and polyamine pathways in Alzheimer disease: A targeted metabolomic and transcriptomic study
Source: PLoS Med. 2020 Jan 24;17(1):e1003012. doi: 10.1371/journal.pmed.1003012 (PMC6980402; doi:10.1371/journal.pmed.1003012)
Supplement: S1 Table — *Indicates that relative measurements were performed for these two metabolites. The values provided represent the average areas of urea and NAA peaks relative to the methionine sulfone internal standard added to the test samples. **Spermine was dropped from the analysis due to above threshold (>30%) missing values. Count/120 denotes the total number of samples in which the metabolite was detected. m/z, mass-to-charge ratio; MT, migration time; NAA, N-acetylaspartate; S/N; signal-to-noise ratio. (DOCX) [file pmed.1003012.s002.docx]

**S1 Table**. **Metabolites studied in this report including their class, ion type, m/z and MT data**

| **(M-H)-** | **Annotation** | **KEGG ID** | **HMDB** | **Count/120** | **Avg pm/mg of brain tissue** | ***m/z*** | **MT** | **S/N** | **Detection Limit (pm/mg)** |
| --- | --- | --- | --- | --- | --- | --- | --- | --- | --- |
| A_0044 | *N*-Acetylglutamic acid | [C00624](http://www.genome.jp/dbget-bin/www_bget?cpd:C00624) | [HMDB01138](http://www.hmdb.ca/metabolites/HMDB01138) | 120 | 153.5 | 188.05682 | 13.2 | 237.6 | 1.9 |
| **(M+H)+** | **Annotation** | **KEGG ID** | **HMDB** | **Count/120** | **Avg pm/mg** | ***m/z*** | **MT** | **S/N** | **S/N** |
| C_0070 | Methionine sulfoxide | [C02989](http://www.genome.jp/dbget-bin/www_bget?cpd:C02989) | [HMDB02005](http://www.hmdb.ca/metabolites/HMDB02005) | 120 | 8 | 166.05368 | 13.7 | 7.7 | 3.1 |
| C_0101 | SDMA | No ID | [HMDB03334](http://www.hmdb.ca/metabolites/HMDB03334) | 120 | 2.6 | 203.15074 | 8.7 | 7.2 | 1.1 |
| C_0111 | Cystathionine | [C00542,C02291](http://www.genome.jp/dbget-bin/www_bget?cpd:C00542) | [HMDB00099](http://www.hmdb.ca/metabolites/HMDB00099) | 120 | 586.5 | 223.0756 | 11.3 | 265.7 | 6.6 |
| C_0152 | Argininosuccinic acid | [C03406](http://www.genome.jp/dbget-bin/www_bget?cpd:C03406) | [HMDB00052](http://www.hmdb.ca/metabolites/HMDB00052) | 120 | 3.5 | 291.13074 | 10.7 | 8.8 | 1.2 |
| C_0164 | *S*-Adenosylhomocysteine | [C00021](http://www.genome.jp/dbget-bin/www_bget?cpd:C00021) | [HMDB00939](http://www.hmdb.ca/metabolites/HMDB00939) | 120 | 13.9 | 385.1297 | 9.9 | 34.6 | 1.2 |
| C_0008 | Ala | [C00041,C00133,C01401](http://www.genome.jp/dbget-bin/www_bget?cpd:C00041) | [HMDB00161,HMDB01310](http://www.hmdb.ca/metabolites/HMDB00161) | 120 | 1916.3 | 90.055 | 10.2 | 397.2 | 14.5 |
| C_0012 | GABA | [C00334](http://www.genome.jp/dbget-bin/www_bget?cpd:C00334) | [HMDB00112](http://www.hmdb.ca/metabolites/HMDB00112) | 120 | 1239 | 104.0709 | 8.6 | 249.8 | 14.9 |
| C_0014 | Choline | [C00114](http://www.genome.jp/dbget-bin/www_bget?cpd:C00114) | [HMDB00097](http://www.hmdb.ca/metabolites/HMDB00097) | 120 | 495.8 | 104.1073 | 7.6 | 567.5 | 2.6 |
| C_0029 | Cys | [C00097,C00736,C00793](http://www.genome.jp/dbget-bin/www_bget?cpd:C00097) | [HMDB00574,HMDB03417](http://www.hmdb.ca/metabolites/HMDB00574) | 120 | 201.7 | 122.0272 | 13 | 229.5 | 2.6 |
| C_0040 | Creatine | [C00300](http://www.genome.jp/dbget-bin/www_bget?cpd:C00300) | [HMDB00064](http://www.hmdb.ca/metabolites/HMDB00064) | 120 | 6494.8 | 132.0769 | 10 | 5611.4 | 3.5 |
| C_0041 | Ornithine | [C00077,C00515,C01602](http://www.genome.jp/dbget-bin/www_bget?cpd:C00077) | [HMDB00214,HMDB03374](http://www.hmdb.ca/metabolites/HMDB00214) | 120 | 50.8 | 133.0974 | 7.6 | 35.9 | 4.2 |
| C_0043 | Asp | [C00049,C00402,C16433](http://www.genome.jp/dbget-bin/www_bget?cpd:C00049) | [HMDB00191,HMDB06483](http://www.hmdb.ca/metabolites/HMDB00191) | 120 | 1786.4 | 134.0449 | 13.3 | 391.0 | 13.7 |
| C_0055 | Spermidine | [C00315](http://www.genome.jp/dbget-bin/www_bget?cpd:C00315) | [HMDB01257](http://www.hmdb.ca/metabolites/HMDB01257) | 120 | 30.8 | 146.1653 | 4.9 | 137.4 | 0.7 |
| C_0056 | Gln | [C00064,C00303,C00819](http://www.genome.jp/dbget-bin/www_bget?cpd:C00064) | [HMDB00641,HMDB03423](http://www.hmdb.ca/metabolites/HMDB00641) | 120 | 4990.4 | 147.0767 | 12.3 | 1435.4 | 10.4 |
| C_0058 | Glu | [C00025,C00217,C00302](http://www.genome.jp/dbget-bin/www_bget?cpd:C00025) | [HMDB00148,HMDB03339](http://www.hmdb.ca/metabolites/HMDB00148) | 120 | 8074.9 | 148.0608 | 12.6 | 2890.6 | 8.4 |
| C_0059 | Met | [C00073,C00855,C01733](http://www.genome.jp/dbget-bin/www_bget?cpd:C00073) | [HMDB00696](http://www.hmdb.ca/metabolites/HMDB00696) | 120 | 218.7 | 150.0587 | 12.3 | 219.9 | 3.0 |
| C_0076 | Arg | [C00062,C00792](http://www.genome.jp/dbget-bin/www_bget?cpd:C00062) | [HMDB00517,HMDB03416](http://www.hmdb.ca/metabolites/HMDB00517) | 120 | 499.4 | 175.1189 | 7.9 | 597.7 | 2.5 |
| C_0078 | Citrulline | [C00327](http://www.genome.jp/dbget-bin/www_bget?cpd:C00327) | [HMDB00904](http://www.hmdb.ca/metabolites/HMDB00904) | 120 | 76.1 | 176.1039 | 12.7 | 46.1 | 4.9 |
| C_0154 | Glutathione (GSSG) divalent | [C00127](http://www.genome.jp/dbget-bin/www_bget?cpd:C00127) | [HMDB03337](http://www.hmdb.ca/metabolites/HMDB03337) | 120 | 165.1 | 307.0841 | 14.1 | 394.1 | 1.3 |
| C_0001 | Urea* | C00086 | HMDB00294 | 120 | 0.56* | 61.04 | 23.6 | 84.1 | 0.02 |
| C_0005 | Putrescine | [C00134](http://www.genome.jp/dbget-bin/www_bget?cpd:C00134) | [HMDB01414](http://www.hmdb.ca/metabolites/HMDB01414) | 119 | 24.6 | 89.1073 | 5.1 | 15.0 | 4.9 |
| C_03166 | *N*-Acetylaspartic acid* | [C01042](http://www.genome.jp/dbget-bin/www_bget?cpd:C01042) | [HMDB00812](http://www.hmdb.ca/metabolites/HMDB00812) | 119 | 6.12 * | 176.055 | 26.49 | 4502.3 | 0.0 |
| C_0165 | *S*-Adenosylmethionine | [C00019](http://www.genome.jp/dbget-bin/www_bget?cpd:C00019) | [HMDB01185](http://www.hmdb.ca/metabolites/HMDB01185) | 119 | 8 | 399.1454 | 7.9 | 24.3 | 1.0 |
| C_0155 | Glutathione (GSH) | [C00051](http://www.genome.jp/dbget-bin/www_bget?cpd:C00051) | [HMDB00125](http://www.hmdb.ca/metabolites/HMDB00125) | 113 | 172.3 | 308.0917 | 15.5 | 302.7 | 1.7 |
| C_0024 | Betaine | [C00719](http://www.genome.jp/dbget-bin/www_bget?cpd:C00719) | [HMDB00043](http://www.hmdb.ca/metabolites/HMDB00043) | 93 | 124.6 | 118.0865 | 13 | 7.0 | 53.6 |
| C_0102 | Spermine | [C00750](http://www.genome.jp/dbget-bin/www_bget?cpd:C00750) | [HMDB01256](http://www.hmdb.ca/metabolites/HMDB01256) | 62 | 7.3 | 203.2234 | 4.9 | 8.1 | 2.7 |

* Indicates that relative measurements were performed for these two metabolites. The values provided represent the average areas of urea and NAA peaks relative to the methionine sulfone internal standard added to the test samples.

** Spermine was dropped from analysis due to above threshold (> 30%) missing values.

S/N; signal:noise

Count/120 denotes the total number of samples in which the metabolite was detected
