# Supplementary material for: Dysregulation of multiple metabolic networks related to brain transmethylation and polyamine pathways in Alzheimer disease: A targeted metabolomic and transcriptomic study
Source: PLoS Med. 2020 Jan 24;17(1):e1003012. doi: 10.1371/journal.pmed.1003012 (PMC6980402; doi:10.1371/journal.pmed.1003012)
Supplement: S2 Table — *Indicates metabolites that could only be relatively quantified. The values provided represent the average areas of urea and NAA peaks relative to the methionine sulfone internal standard added to the test samples. AD, Alzheimer disease; ASY, asymptomatic AD; CB, cerebellum; CE-TOFMS, capillary electrophoresis time-of-flight mass spectrometry; CN, control; ITG, inferior temporal gyrus; MFG, medial frontal gyrus; NAA, N-acetylaspartate. (DOCX) [file pmed.1003012.s003.docx]

**S2 Table. Regional brain tissue concentrations (picomoles per milligram of brain tissue) of metabolites assayed by capillary electrophoresis time-of-flight mass spectrometry (CE-TOFMS)**

|  | ITG | | | MFG | | | CB | | |
| --- | --- | --- | --- | --- | --- | --- | --- | --- | --- |
|  | **AD** | **ASY** | **CN** | **AD** | **ASY** | **CN** | **AD** | **ASY** | **CN** |
| Alanine | 2147.727 | 2010.427 | 1916.502 | 1670.967 | 1650.186 | 1545.128 | 2146.387 | 2028.784 | 2317.791 |
| Arginine | 553.024 | 527.705 | 447.339 | 406.453 | 389.552 | 333.975 | 622.702 | 583.141 | 764.147 |
| Aspartate | 1697.2 | 2068.507 | 2224.454 | 1351.105 | 1712.173 | 1784.718 | 1748.343 | 1695.375 | 2198.271 |
| Betaine | 85.815 | 86.191 | 130.04 | 87.252 | 102.212 | 117.436 | 103.838 | 91.506 | 124.507 |
| Choline | 375.389 | 470.349 | 756.151 | 315.868 | 413.477 | 645.185 | 460.798 | 507.47 | 681.202 |
| Citrulline | 76.187 | 91.54 | 85.031 | 42.859 | 58.758 | 79.677 | 79.323 | 71.403 | 139.452 |
| Creatine | 6216.002 | 6263.294 | 5962.509 | 5218.692 | 5163.396 | 4731.436 | 8697.022 | 8385.721 | 8545.685 |
| Cysteine | 382.245 | 189.753 | 111.81 | 254.138 | 87.557 | 63.224 | 276.626 | 157.304 | 276.986 |
| GABA | 1107.401 | 1447.963 | 1471.191 | 1047.736 | 1247.565 | 1286.138 | 1142.701 | 1185.362 | 1383.455 |
| Glutamine | 5200.442 | 5499.598 | 4693.919 | 4291.165 | 4998.839 | 3778.312 | 5610.641 | 5286.284 | 5045.883 |
| Glutamate | 8630.782 | 9020.212 | 8729.433 | 7086.272 | 7283.645 | 6536.885 | 8513.834 | 8287.258 | 8540.24 |
| Glutathione (GSH) | 276.934 | 161.552 | 83.315 | 253.26 | 115.45 | 71.197 | 209.557 | 98.551 | 143.476 |
| Glutathione (GSSG) | 136.749 | 183.447 | 134.49 | 160.398 | 186.059 | 145.453 | 173.495 | 204.835 | 145.007 |
| Methionine | 250.683 | 217.249 | 191.727 | 163.728 | 159.821 | 144.644 | 270.574 | 297.393 | 352.212 |
| Ornithine | 37.577 | 47.926 | 65.795 | 30.605 | 36.867 | 64.858 | 53.131 | 66.33 | 81.11 |
| Putrescine | 26.717 | 31.713 | 30.557 | 26.758 | 30.875 | 26.797 | 10.827 | 14.97 | 17.619 |
| S-Adenosylmethionine | 10.472 | 8.872 | 5.873 | 8.616 | 7.29 | 5.153 | 7.71 | 8.61 | 8.244 |
| Spermidine | 44.337 | 46.687 | 27.504 | 32.917 | 39.346 | 25.121 | 19.041 | 17.556 | 13.544 |
| N-Acetyl glutamic acid | 118.344 | 168.725 | 177.242 | 106.604 | 140.01 | 137.356 | 183.236 | 189.064 | 223.728 |
| Cystathionine | 569.002 | 673.409 | 947.71 | 913.309 | 801.43 | 528.764 | 283.363 | 201.61 | 255.227 |
| S-Adenosylhomocysteine | 14.268 | 14.142 | 13.649 | 13.014 | 11.961 | 9.198 | 17.141 | 16.23 | 16.321 |
| Methionine sulfoxide | 6.158 | 8.926 | 9.042 | 5.794 | 6.532 | 10.118 | 8.782 | 5.689 | 12.445 |
| SDMA | 2.661 | 3.077 | 2.24 | 2.195 | 2.822 | 1.794 | 2.655 | 3.287 | 2.897 |
| Argininosuccinic acid | 2.836 | 6.202 | 3.144 | 1.622 | 5.697 | 2.218 | 3.292 | 3.09 | 4.207 |
| N-Acetyl aspartic acid | 0.117 | 0.145 | 0.157 | 0.121 | 0.122 | 0.123 | 0.117 | 0.11 | 0.122 |
| Urea | 0.648 | 0.592 | 0.464 | 0.581 | 0.598 | 0.433 | 0.572 | 0.638 | 0.369 |

AD: Alzheimer’s disease, ASY: asymptomatic Alzheimer’s disease), CN: control. ITG: inferior temporal gyrus, MFG: medial frontal gyrus, CB: cerebellum.

*indicates metabolites that could only be relatively quantified. The values provided represent the average areas of urea and NAA peaks relative to the methionine sulfone internal standard added to the test samples.
