# Supplementary material for: Dysregulation of multiple metabolic networks related to brain transmethylation and polyamine pathways in Alzheimer disease: A targeted metabolomic and transcriptomic study
Source: PLoS Med. 2020 Jan 24;17(1):e1003012. doi: 10.1371/journal.pmed.1003012 (PMC6980402; doi:10.1371/journal.pmed.1003012)
Supplement: S3 Table — p-Value indicates significance after FDR correction for 26 comparisons. Group differences were tested using linear mixed-effects models for each metabolite category. Bon, Bonferroni; CB, cerebellum, FDR, false discovery rate. (DOCX) [file pmed.1003012.s004.docx]

**S3 Table. Group differences in the Cerebellum (CB)**

|  | **Group differences** | |
| --- | --- | --- |
|  | **FDR (26)** | **Bon (26)** |
| Betaine | 0.6169 | 1 |
| Choline | 0.1014 | 1 |
| Creatine | 0.8494 | 1 |
| Met | 0.1955 | 1 |
| S-Adenosylmethionine (SAM) | 0.5228 | 1 |
| Methionine sulfoxide | 0.6983 | 1 |
| S-Adenosylhomocysteine (SAH) | 0.5617 | 1 |
| SDMA | 0.3796 | 1 |
| Cysteine | 0.5823 | 1 |
| Glutathione (GSH) | 0.0751 | 1 |
| Glutathione (GSSG) | 0.5729 | 1 |
| Cystathionine | 0.5649 | 1 |
| Putrescine | 0.0683 | 1 |
| Spermidine | 0.5604 | 1 |
| Urea | 0.051 | 1 |
| Arginine | 0.2935 | 1 |
| Citrulline | 0.2984 | 1 |
| Ornithine | 0.1625 | 1 |
| Argininosuccinic acid | 0.3063 | 1 |
| N-Acetylglutamic acid (NAG) | 0.0537 | 1 |
| Alanine | 0.648 | 1 |
| Aspartate | 0.1841 | 1 |
| Glutamine | 0.3978 | 1 |
| Glutamate | 0.9367 | 1 |
| N-Acetylaspartic acid (NAA) | 0.7489 | 1 |
| GABA | 0.0705 | 1 |

CB: cerebellum, FDR: False Discovery Rate, Bon: Bonferroni

P-value indicates significance after FDR correction for 26 comparisons.

Group difference were tested using linear mixed-effects models for each metabolite category.
