# Supplementary material for: Dysregulation of multiple metabolic networks related to brain transmethylation and polyamine pathways in Alzheimer disease: A targeted metabolomic and transcriptomic study
Source: PLoS Med. 2020 Jan 24;17(1):e1003012. doi: 10.1371/journal.pmed.1003012 (PMC6980402; doi:10.1371/journal.pmed.1003012)
Supplement: S4 Table — aCompiled from open source references, including GeneCards (genecards.org) and Wikipedia (wikipedia.org). Note: gene expression significance was calculated in the hippocampus and ERC regions; genes represented in the table were significantly different between AD and CN individuals in at least one of the two regions. AD, Alzheimer disease; CN, control; ERC, entorhinal cortex. (DOCX) [file pmed.1003012.s005.docx]

**S4 Table. Role and clinical significance of genes differentially expressed in AD compared to CN**

| Gene | Role ^a^ | Clinical Significance (if previously reported) |
| --- | --- | --- |
| Methionine Cycle | | |
| AHCY | Catalyzes reversible conversion of SAH to adenosine and homocysteine | Suppression of age-dependent SAH accumulation is associated with increased life- and health span in drosophila [1]. Hyperhomocysteinemia may be risk factor for AD [2], [3], [4].  S-adenosylhomocysteine hydrolase inhibitors may be novel therapeutic agents in AD [5]. |
| BHMT2 | Transfers methyl group from betaine to homocysteine | Hyperhomocysteinemia may be risk factor for AD [2], [3], [4] |
| CHDH | Step 1 of the pathway that converts choline to betaine aldehyde | Increased gene expression of CHDH in brains of bipolar disorder (BPD) patients and increased risk of BPD associated with rs9836592 SNP in the CHDH gene [6] |
| MAT1A | Transfers adenosyl moiety of ATP to methionine to form SAM | SNPs in MAT1A are associated with longevity and cognitive ability during aging [7] |
| MTHFD1 | Encodes a trifunctional folate-metabolizing enzyme, C1-tetrahydrofolate synthetase catalyzing ATP-dependent conversion of formate and THF to 5, 10-methylene tetrahydrofolate | The MTHFD1 G1958A SNP results in a thermolabile protein with reduced C1-tetrahydrofolate synthetase activity and is associated with increased maternal risk of neural tube defects [8] |
| MTR | Catalyzes the final step in methionine biosynthesis | Multiple polymorphisms are associated with AD and Parkinson’s disease [9], [10] [11]; |
| SHMT1 | Cytosolic form; catalyzes reversible conversion of serine and tetrahydrofolate to glycine and 5,10-methylene tetrahydrofolate |  |
| SHMT2 | Mitochondrial form; catalyzes reversible conversion of serine and tetrahydrofolate to glycine and 5,10-methylene tetrahydrofolate | Increased gene expression of SHMT2 glioblastoma multiforme (GBM) [12] |
| Transsulfuration and Glutathione Synthesis | | |
| CTH | Converts cystathionine into cysteine | A common mutation (1364T/T) in CTH is associated with hyperhomocysteinemia [13] which is a risk factor for AD [2], [3], [4]) |
| DLD | Member of the Glycine cleavage system (GCS); one of 4 sequential enzymes to produce a folate derivative that transfers one‑carbon units in cellular reactions | DLD gene variant is associated with schizophrenia [14] |
| GSS | Catalyzes the second step of glutathione biosynthesis | Alterations in brain GSH levels are associated with AD, modulation of GSH levels may be a novel therapeutic strategy in AD [15], [16]. |
| Polyamine Synthesis and Catabolism | | |
| SAT1 | Rate-limiting enzyme in polyamine catabolic pathway | One of the most consistently implicated genes in major depression and suicide [17] |
| SMOX | Catalyzes the oxidation of spermine to spermidine | Mediator of neuroinflammation in cerebral ischemia and is associated with glutamate related excitotoxicity [18], [19] |
| SRM | Catalyzes final step of spermidine biosynthesis | Modulates neuronal survival and memory consolidation [20] |
| Urea Cycle | | |
| ARG2 | Catalyzes the hydrolysis of arginine to ornithine and urea | Rare ARG2 allele rs742869 is associated with increased risk of AD [21] |
| ASL | Carries out the final step of arginine synthesis |  |
| ASS1 | Catalyzes the rate-limiting step in arginine synthesis |  |
| GATM | Controls first step in production of creatine from arginine and glycine |  |
| ODC1 | Catalyzes the rate-limiting reaction in polyamine biosynthesis | The ODC1 gene has been implicated in neoplasia including neuroblastoma and ODC1 inhibitors are under investigation as cancer chemotherapy agents. [22] |
| OAT | Key mitochondrial enzyme converting arginine and ornithine into the major excitatory and inhibitory neurotransmitters- glutamate and GABA respectively. |  |
| Glutamate-Aspartate Metabolism | | |
| ASNS | Converts aspartate and glutamine to asparagine and glutamate | Mutations cause a distinct neurodevelopmental disorder characterized by congenital microcephaly and encephalopathy cerebral atrophy [23],[24] |
| GLS | Synthesizes neurotransmitter pool of glutamate (kidney type) | GLS activity correlated with brain tumor growth rate and malignancy [25] |
| GLS2 | Synthesizes neurotransmitter pool of glutamate (liver type) | Affects neurite outgrowth [26] |
| GOT2 | Plays role in amino acid metabolism, urea and tricarboxylic acid cycles, malate-aspartate shuttle | Gene expression of GOT2 has previously been reported to be reduced in AD brain [27] |
| MDH1 | Catalyzes reversible oxidation of malate to oxaloacetate, cytosolic isoform | Reduced gene expression of MDH1 in dorsolateral prefrontal cortex (DLPFC) in schizophrenia [28]; increased CSF levels of MDH1 in sporadic Creutzfeldt-Jakob disease [29] |
| MDH2 | Catalyzes reversible oxidation of reversible oxidation of malate to oxaloacetate, mitochondrial isoform | MDH2 mutations cause early onset encephalopathy [30] |
| Neurotransmitter Metabolism | | |
| ALDH1A1 | Second enzyme of major oxidative pathway of alcohol metabolism, cytosolic isozyme | Decreased in the substantia nigra in Parkinson's disease patients and may be involved in AD neurodegeneration through role in detoxification of toxic aldehydes [31], [32] |
| FH | Enzymatic component of TCA cycle, catalyzes formation of L-malate from fumarate | FH mutations cause autosomal recessive fumaric aciduria in early childhood characterized by seizures, developmental delay, and mental retardation [33] |
| KYAT3 | Metabolizes tryptophan by converting kynurenine to kynurenic acid | Kynurenic acid levels (in blood and brain) correlate with cognitive decline and imminent mortality [34], and changes in kynurenic acid levels may affect acetylcholine neurotransmission [35] |
| MAOB | Plays role in the catabolism of neuroactive and vasoactive amines in CNS and peripheral tissues, degrades dopamine | MAO-B is associated with gamma-secretase and regulates neuronal amyloid beta-peptide levels [36], increased MAO-B activity is seen in plaque-associated astrocytes in AD [37] |
| ME1 | Generates NADPH for fatty acid biosynthesis, links the glycolytic and citric acid cycles | Declines in aged cerebellum and aging hippocampus in mice [38] |
| SLC25A12 | Regulates exchange of aspartate for glutamate across the inner mitochondrial membrane | Polymorphisms may be associated with autism [39], reduced expression in neuroinflammation [40] |

^a^ Compiled from open source references, including GeneCards (genecards.org) and Wikipedia (wikipedia.org).

AD: Alzheimer’s disease; CN: control

Note: gene expression significance was calculated in the hippocampus and entorhinal cortex regions; genes represented in the table were significantly different between Alzheimer’s disease and control individuals in at least one of the two regions

1. Parkhitko AA, Binari R, Zhang N, Asara JM, Demontis F, Perrimon N. Tissue-specific down-regulation of S-adenosyl-homocysteine via suppression of dAhcyL1/dAhcyL2 extends health span and life span in Drosophila. Genes Dev. 2016;30(12):1409-22. Epub 2016/06/18. doi: 10.1101/gad.282277.116. PubMed PMID: 27313316; PubMed Central PMCID: PMCPMC4926864.

2. Nazef K, Khelil M, Chelouti H, Kacimi G, Bendini M, Tazir M, et al. Hyperhomocysteinemia is a risk factor for Alzheimer's disease in an Algerian population. Arch Med Res. 2014;45(3):247-50. Epub 2014/03/25. doi: 10.1016/j.arcmed.2014.03.001. PubMed PMID: 24656904.

3. Gallucci M, Zanardo A, De Valentin L, Vianello A. Homocysteine in Alzheimer disease and vascular dementia. Arch Gerontol Geriatr Suppl. 2004;(9):195-200. Epub 2004/06/23. doi: 10.1016/j.archger.2004.04.027. PubMed PMID: 15207414.

4. Ientile R, Curro M, Ferlazzo N, Condello S, Caccamo D, Pisani F. Homocysteine, vitamin determinants and neurological diseases. Front Biosci (Schol Ed). 2010;2:359-72. Epub 2009/12/29. PubMed PMID: 20036953.

5. Converso A, Hartingh T, Fraley ME, Garbaccio RM, Hartman GD, Huang SY, et al. Adenosine analogue inhibitors of S-adenosylhomocysteine hydrolase. Bioorg Med Chem Lett. 2014;24(12):2737-40. Epub 2014/05/13. doi: 10.1016/j.bmcl.2014.04.034. PubMed PMID: 24813734.

6. Chang H, Li L, Peng T, Grigoroiu-Serbanescu M, Bergen SE, Landen M, et al. Identification of a Bipolar Disorder Vulnerable Gene CHDH at 3p21.1. Mol Neurobiol. 2017;54(7):5166-76. Epub 2016/08/27. doi: 10.1007/s12035-016-0041-x. PubMed PMID: 27562178.

7. Lopez LM, Harris SE, Luciano M, Liewald D, Davies G, Gow AJ, et al. Evolutionary conserved longevity genes and human cognitive abilities in elderly cohorts. Eur J Hum Genet. 2012;20(3):341-7. Epub 2011/11/03. doi: 10.1038/ejhg.2011.201. PubMed PMID: 22045296; PubMed Central PMCID: PMCPMC3283186.

8. Jiang J, Zhang Y, Wei L, Sun Z, Liu Z. Association between MTHFD1 G1958A polymorphism and neural tube defects susceptibility: a meta-analysis. PLoS One. 2014;9(6):e101169. Epub 2014/07/01. doi: 10.1371/journal.pone.0101169. PubMed PMID: 24977710; PubMed Central PMCID: PMCPMC4076264.

9. Dorszewska J, Florczak J, Rozycka A, Kempisty B, Jaroszewska-Kolecka J, Chojnacka K, et al. Oxidative DNA damage and level of thiols as related to polymorphisms of MTHFR, MTR, MTHFD1 in Alzheimer's and Parkinson's diseases. Acta Neurobiol Exp (Wars). 2007;67(2):113-29. Epub 2007/08/19. PubMed PMID: 17691219.

10. Coppede F, Tannorella P, Pezzini I, Migheli F, Ricci G, Caldarazzo lenco E, et al. Folate, homocysteine, vitamin B12, and polymorphisms of genes participating in one-carbon metabolism in late-onset Alzheimer's disease patients and healthy controls. Antioxid Redox Signal. 2012;17(2):195-204. Epub 2011/11/01. doi: 10.1089/ars.2011.4368. PubMed PMID: 22034983.

11. Rozycka A, Jagodzinski PP, Kozubski W, Lianeri M, Dorszewska J. Homocysteine Level and Mechanisms of Injury in Parkinson's Disease as Related to MTHFR, MTR, and MTHFD1 Genes Polymorphisms and L-Dopa Treatment. Curr Genomics. 2013;14(8):534-42. Epub 2014/02/18. doi: 10.2174/1389202914666131210210559. PubMed PMID: 24532985; PubMed Central PMCID: PMCPMC3924248.

12. Kim D, Fiske BP, Birsoy K, Freinkman E, Kami K, Possemato RL, et al. SHMT2 drives glioma cell survival in ischaemia but imposes a dependence on glycine clearance. Nature. 2015;520(7547):363-7. Epub 2015/04/10. doi: 10.1038/nature14363. PubMed PMID: 25855294; PubMed Central PMCID: PMCPMC4533874.

13. Roman GC, Mancera-Paez O, Bernal C. Epigenetic Factors in Late-Onset Alzheimer's Disease: MTHFR and CTH Gene Polymorphisms, Metabolic Transsulfuration and Methylation Pathways, and B Vitamins. Int J Mol Sci. 2019;20(2). Epub 2019/01/17. doi: 10.3390/ijms20020319. PubMed PMID: 30646578; PubMed Central PMCID: PMCPMC6359124.

14. Yoshikawa A, Nishimura F, Inai A, Eriguchi Y, Nishioka M, Takaya A, et al. Mutations of the glycine cleavage system genes possibly affect the negative symptoms of schizophrenia through metabolomic profile changes. Psychiatry Clin Neurosci. 2018;72(3):168-79. Epub 2017/12/13. doi: 10.1111/pcn.12628. PubMed PMID: 29232014.

15. Peter C, Braidy N, Zarka M, Welch J, Bridge W. Therapeutic approaches to modulating glutathione levels as a pharmacological strategy in Alzheimer's disease. Curr Alzheimer Res. 2015;12(4):298-313. Epub 2015/03/04. PubMed PMID: 25731620.

16. Saharan S, Mandal PK. The emerging role of glutathione in Alzheimer's disease. J Alzheimers Dis. 2014;40(3):519-29. Epub 2014/02/06. doi: 10.3233/JAD-132483. PubMed PMID: 24496077.

17. Pantazatos SP, Andrews SJ, Dunning-Broadbent J, Pang J, Huang YY, Arango V, et al. Isoform-level brain expression profiling of the spermidine/spermine N1-Acetyltransferase1 (SAT1) gene in major depression and suicide. Neurobiol Dis. 2015;79:123-34. Epub 2015/05/12. doi: 10.1016/j.nbd.2015.04.014. PubMed PMID: 25959060; PubMed Central PMCID: PMCPMC4834874.

18. Pietropaoli S, Leonetti A, Cervetto C, Venturini A, Mastrantonio R, Baroli G, et al. Glutamate Excitotoxicity Linked to Spermine Oxidase Overexpression. Mol Neurobiol. 2018;55(9):7259-70. Epub 2018/02/06. doi: 10.1007/s12035-017-0864-0. PubMed PMID: 29397558.

19. Fan J, Chen M, Wang X, Tian Z, Wang J, Fan D, et al. Targeting Smox is neuroprotective and ameliorates brain inflammation in cerebral ischemia/reperfusion rats. Toxicol Sci. 2018. Epub 2018/12/24. doi: 10.1093/toxsci/kfy300. PubMed PMID: 30576531.

20. Guerra GP, Rubin MA, Mello CF. Modulation of learning and memory by natural polyamines. Pharmacol Res. 2016;112:99-118. Epub 2016/03/27. doi: 10.1016/j.phrs.2016.03.023. PubMed PMID: 27015893.

21. Hansmannel F, Sillaire A, Kamboh MI, Lendon C, Pasquier F, Hannequin D, et al. Is the urea cycle involved in Alzheimer's disease? J Alzheimers Dis. 2010;21(3):1013-21. Epub 2010/08/10. doi: 10.3233/JAD-2010-100630. PubMed PMID: 20693631; PubMed Central PMCID: PMCPMC2945690.

22. Hogarty MD, Norris MD, Davis K, Liu X, Evageliou NF, Hayes CS, et al. ODC1 is a critical determinant of MYCN oncogenesis and a therapeutic target in neuroblastoma. Cancer Res. 2008;68(23):9735-45. Epub 2008/12/03. doi: 10.1158/0008-5472.CAN-07-6866. PubMed PMID: 19047152; PubMed Central PMCID: PMCPMC2596661.

23. Ruzzo EK, Capo-Chichi JM, Ben-Zeev B, Chitayat D, Mao H, Pappas AL, et al. Deficiency of asparagine synthetase causes congenital microcephaly and a progressive form of encephalopathy. Neuron. 2013;80(2):429-41. Epub 2013/10/22. doi: 10.1016/j.neuron.2013.08.013. PubMed PMID: 24139043; PubMed Central PMCID: PMCPMC3820368.

24. Ben-Salem S, Gleeson JG, Al-Shamsi AM, Islam B, Hertecant J, Ali BR, et al. Asparagine synthetase deficiency detected by whole exome sequencing causes congenital microcephaly, epileptic encephalopathy and psychomotor delay. Metab Brain Dis. 2015;30(3):687-94. Epub 2014/09/18. doi: 10.1007/s11011-014-9618-0. PubMed PMID: 25227173; PubMed Central PMCID: PMCPMC4915861.

25. Campos-Sandoval JA, Martin-Rufian M, Cardona C, Lobo C, Penalver A, Marquez J. Glutaminases in brain: Multiple isoforms for many purposes. Neurochem Int. 2015;88:1-5. Epub 2015/04/04. doi: 10.1016/j.neuint.2015.03.006. PubMed PMID: 25837287.

26. Niklison-Chirou MV, Killick R, Knight RA, Nicotera P, Melino G, Agostini M. How Does p73 Cause Neuronal Defects? Mol Neurobiol. 2016;53(7):4509-20. Epub 2015/08/13. doi: 10.1007/s12035-015-9381-1. PubMed PMID: 26266644.

27. Puthiyedth N, Riveros C, Berretta R, Moscato P. Identification of Differentially Expressed Genes through Integrated Study of Alzheimer's Disease Affected Brain Regions. PLoS One. 2016;11(4):e0152342. Epub 2016/04/07. doi: 10.1371/journal.pone.0152342. PubMed PMID: 27050411; PubMed Central PMCID: PMCPMC4822961.

28. Vawter MP, Shannon Weickert C, Ferran E, Matsumoto M, Overman K, Hyde TM, et al. Gene expression of metabolic enzymes and a protease inhibitor in the prefrontal cortex are decreased in schizophrenia. Neurochem Res. 2004;29(6):1245-55. Epub 2004/06/05. PubMed PMID: 15176481.

29. Schmitz M, Llorens F, Pracht A, Thom T, Correia A, Zafar S, et al. Regulation of human cerebrospinal fluid malate dehydrogenase 1 in sporadic Creutzfeldt-Jakob disease patients. Aging (Albany NY). 2016;8(11):2927-35. Epub 2016/11/18. doi: 10.18632/aging.101101. PubMed PMID: 27852982; PubMed Central PMCID: PMCPMC5191879.

30. Ait-El-Mkadem S, Dayem-Quere M, Gusic M, Chaussenot A, Bannwarth S, Francois B, et al. Mutations in MDH2, Encoding a Krebs Cycle Enzyme, Cause Early-Onset Severe Encephalopathy. Am J Hum Genet. 2017;100(1):151-9. Epub 2016/12/19. doi: 10.1016/j.ajhg.2016.11.014. PubMed PMID: 27989324; PubMed Central PMCID: PMCPMC5223029.

31. Grunblatt E, Riederer P. Aldehyde dehydrogenase (ALDH) in Alzheimer's and Parkinson's disease. J Neural Transm (Vienna). 2016;123(2):83-90. Epub 2014/10/10. doi: 10.1007/s00702-014-1320-1. PubMed PMID: 25298080.

32. Chen CH, Joshi AU, Mochly-Rosen D. The Role of Mitochondrial Aldehyde Dehydrogenase 2 (ALDH2) in Neuropathology and Neurodegeneration. Acta Neurol Taiwan. 2016;25(4):111-23. Epub 2017/04/07. PubMed PMID: 28382610.

33. Ezgu F, Krejci P, Wilcox WR. Mild clinical presentation and prolonged survival of a patient with fumarase deficiency due to the combination of a known and a novel mutation in FH gene. Gene. 2013;524(2):403-6. Epub 2013/04/25. doi: 10.1016/j.gene.2013.03.026. PubMed PMID: 23612258.

34. Stone TW, Darlington LG. The kynurenine pathway as a therapeutic target in cognitive and neurodegenerative disorders. Br J Pharmacol. 2013;169(6):1211-27. Epub 2013/05/08. doi: 10.1111/bph.12230. PubMed PMID: 23647169; PubMed Central PMCID: PMCPMC3831703.

35. Han Q, Cai T, Tagle DA, Li J. Structure, expression, and function of kynurenine aminotransferases in human and rodent brains. Cell Mol Life Sci. 2010;67(3):353-68. Epub 2009/10/15. doi: 10.1007/s00018-009-0166-4. PubMed PMID: 19826765; PubMed Central PMCID: PMCPMC2867614.

36. Schedin-Weiss S, Inoue M, Hromadkova L, Teranishi Y, Yamamoto NG, Wiehager B, et al. Monoamine oxidase B is elevated in Alzheimer disease neurons, is associated with gamma-secretase and regulates neuronal amyloid beta-peptide levels. Alzheimers Res Ther. 2017;9(1):57. Epub 2017/08/03. doi: 10.1186/s13195-017-0279-1. PubMed PMID: 28764767; PubMed Central PMCID: PMCPMC5540560.

37. Saura J, Luque JM, Cesura AM, Da Prada M, Chan-Palay V, Huber G, et al. Increased monoamine oxidase B activity in plaque-associated astrocytes of Alzheimer brains revealed by quantitative enzyme radioautography. Neuroscience. 1994;62(1):15-30. Epub 1994/09/01. PubMed PMID: 7816197.

38. Uittenbogaard M, Chiaramello A. Expression of the basic Helix-Loop-Helix ME1 E-protein during development and aging of the murine cerebellum. Neurosci Lett. 1999;274(3):191-4. Epub 1999/11/05. PubMed PMID: 10548422.

39. Aoki Y, Cortese S. Mitochondrial Aspartate/Glutamate Carrier SLC25A12 and Autism Spectrum Disorder: a Meta-Analysis. Mol Neurobiol. 2016;53(3):1579-88. Epub 2015/02/11. doi: 10.1007/s12035-015-9116-3. PubMed PMID: 25663199.

40. Menga A, Iacobazzi V, Infantino V, Avantaggiati ML, Palmieri F. The mitochondrial aspartate/glutamate carrier isoform 1 gene expression is regulated by CREB in neuronal cells. Int J Biochem Cell Biol. 2015;60:157-66. Epub 2015/01/20. doi: 10.1016/j.biocel.2015.01.004. PubMed PMID: 25597433; PubMed Central PMCID: PMCPMC4344217.
